# Vertical Interfacial Engineering in Two-Step-Processed Perovskite Films Enabled by Dual-Interface Modification for High-Efficiency p-i-n Solar Cells

**DOI:** 10.1007/s40820-025-02010-w

**Published:** 2026-01-05

**Authors:** Wenhao Zhou, Heng Liu, Haiyan Li, Weihai Zhang, Hui Li, Xia Zhou, Rouxi Chen, Wenjun Zhang, Tingting Shi, Antonio Abate, Hsing-Lin Wang

**Affiliations:** 1 School of New Energy, Ningbo University of Technology, Ningbo, 315211 People’s Republic of China; 2 Department of Materials Science and Engineering, Southern University of Science and Technology, Shenzhen, 518055 People’s Republic of China; 3 Institute of Materials, Henan Academy of Sciences, Zhengzhou, 450046 People’s Republic of China; 4 Siyuan Laboratory, Guangzhou Key Laboratory of Vacuum Coating Technologies and New Energy Materials, Department of Physics, Jinan University, Guangzhou, 510632 People’s Republic of China; 5 School of Innovation and Entrepreneurship, Southern University of Science and Technology, Shenzhen, 518055 People’s Republic of China

**Keywords:** Vertical interfacial engineering, Interface modification, Energy-level modulation, Nickle oxide, Two-step procession

## Abstract

Two-step-processed (TSP) inverted p-i-n perovskite solar cells (PSCs) have demonstrated significant promise in tandem applications. However, the power conversion efficiency (PCE) of TSP p-i-n PSCs rarely exceeds 24%. Here, we demonstrate that TSP perovskite films exhibit a vertically gradient distribution of residual PbI_2_ clusters, which form Schottky heterojunctions with the perovskite, leading to substantial interfacial energy-level mismatches within NiO_x_-based TSP p-i-n PSCs. These limitations were effectively addressed via a vertical interfacial engineering enabled by dual-interface modification incorporating tin trifluoromethanesulfonate (Sn(OTF)_2_) and 4-Fluorophenylethylamine chloride (F-PEA) at the NiO_x_/perovskite and perovskite/C60 interfaces, respectively. The functional Sn(OTF)_2_ not only enhances the conductivity of NiO_x_ films but also suppresses ion migration, while inducing the formation of a Pb-Sn mixed perovskite interlayer that precisely regulates the energy level at the NiO_x_/perovskite interface. Complementally, F-PEA post-treatment effectively converts surface residual PbI_2_ clusters into a 2D perovskite capping layer, which simultaneously passivates surface defects and enhances energy-level alignment at the perovskite/C60 interface. Consequently, the optimized NiO_x_-based TSP p-i-n PSCs achieve a notable PCE of 25.6% with superior operational stability. This study elucidates the underlying mechanisms limiting the efficiency of TSP p-i-n PSCs, while establishing design principles for these devices targeting 26% efficiency.

**Supplementary Information:**

The online version contains supplementary material available at 10.1007/s40820-025-02010-w.

## Highlights

- A vertical interfacial engineering strategy via dual-interface modification (Sn(OTF)_2_ at NiO_x_/perovskite, 4-Fluorophenylethylamine chloride (F-PEA) at perovskite/C60) solves energy-level mismatches in two-step-processed (TSP) p-i-n PSCs, boosting PCE to 25.6%.
- Sn(OTF)_2_ enhances NiO_x_ conductivity, suppresses ion migration, and forms a Pb-Sn perovskite interlayer; F-PEA eliminates PbI_2_, forming a 2D capping layer for defect passivation.
- Optimized NiO_x_-based TSP p-i-n PSCs retain 84% initial power conversion efficiency after 720-h light illumination, providing design principles for 26%-efficiency devices.

**Supplementary Information:**

The online version contains supplementary material available at 10.1007/s40820-025-02010-w.

## Graphical abstract

**Figure:**
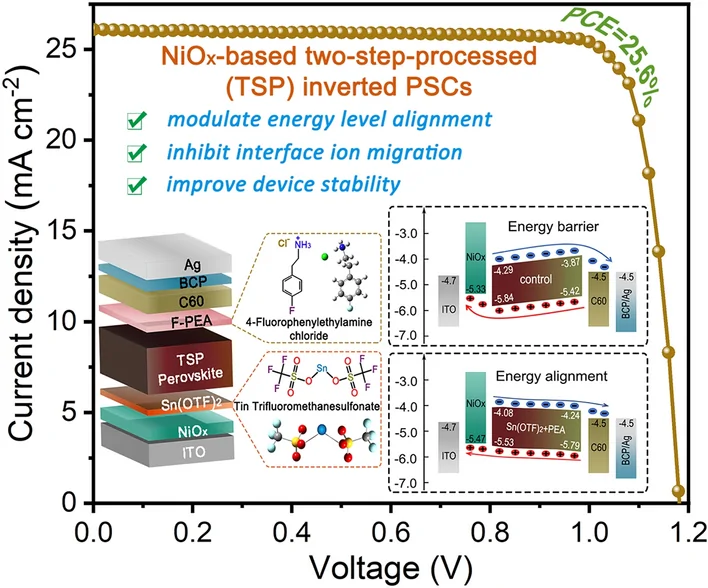

## Introduction

Perovskite solar cells (PSCs) have garnered significant global research interest over the past decade owing to their exceptional advantages, including cost-effective fabrication, high device efficiency, and superior defect tolerance [1, 2]. Through innovations in material design and device engineering, the power conversion efficiency (PCE) of PSCs has reached certified record values of 27.0% in single-junction configurations and 34.6% in perovskite-silicon tandem architectures, positioning them as a leading candidate for next-generation photovoltaic technologies [3–5]. Among strategies for advancing PSCs, the inverted p-i-n architecture has attracted considerable attention due to its negligible hysteresis, low-temperature processability, high stability, and low parasitic absorption. Such features render this architecture particularly suitable for flexible devices and monolithic perovskite-silicon tandem solar cells.

Within p-i-n device architectures, perovskite films are typically deposited on a hole transport layer (HTL) such as poly(3,4-ethylenedioxythiophene)/polystyrene sulfonate (PEDOT/PSS) and poly[bis(4-phenyl)(2,4,6-trimethylphenyl)amine] (PTAA). However, PEDOT/PSS suffers from inherent hydrolytic and UV-induced degradation, whereas PTAA exhibits poor surface wettability, posing substantial manufacturing challenges [6–8]. In comparison, nickel oxide (NiO_x_), an inorganic wide-bandgap semiconductor, has emerged as a promising alternative hole transport material (HTM) for perovskite photovoltaics owing to its combination of exceptional stability, efficient charge carrier transport, and low-cost processing [9–11]. Yu et al. developed H_2_O_2_-engineered NiO_x_ as the HTM, demonstrating that H_2_O_2_ addition enhances NiO_x_ films’ conductivity and generates abundant surface hydroxyl groups for improved surface wettability. This approach yielded NiO_x_-based p-i-n PSCs with a certified PCE of 25.2% [12]. Most recently, Liu et al. reported a molecular hybridization strategy through co-assembling 4,4’,4’’-nitrilotribenzoic acid (NA) with [4-(3,6-dimethyl-9H-carbazol-9-yl)butyl]phosphonic acid (Me-4PACz) to improve the quality of NiO_x_/perovskite interface [13]. They indicated that the molecular hybridization of Me-4PACz with NA significantly promotes charge carrier extraction from the perovskite to NiO_x_ layer. Consequently, the resulting PSCs achieved a certified steady-state efficiency of 26.54%. This impressive PCE value further validates the potential of NiO_x_ as an HTM for p-i-n perovskite photovoltaics.

However, it should be noted that most high-efficiency p-i-n PSCs fabricated on NiO_x_ hole transport layer (HTL) are mainly processed by the one-step deposition method. Although the two-step sequential deposition method is widely regarded as more reproducible and readily scalable, the efficiency of two-step-processed (TSP) p-i-n devices rarely exceeds 24%, demonstrating significantly inferior performance when compared to their one-step counterparts [14, 15]. The fundamental mechanisms underlying the suboptimal performance are primarily attributed to the uncontrollable reaction between pre-deposited PbI_2_ and ammonium salts during the two-step sequential deposition process, generating crystalline phase heterogeneity, such as residual PbI_2_ clusters at the bottom, within the bulk, and on the surface of the perovskite, which deteriorate device efficiency and stability [16, 17]. To address these challenges, researchers have developed various strategies including additive engineering [18], crystallization modulation [19], and particularly focusing on buried interface engineering [20, 21] to reduce residual PbI_2_ cluster formation while simultaneously improving the crystallinity of perovskite films. For example, Zhang et al. introduced a thin layer of CsBr at the top surface of NiO_x_ before PbI_2_ deposition [22]. They suggest that the buried modification of CsBr not only passivated NiO_x_ surface defects to reduce recombination but also regulated the crystal growth orientation of the upper PbI_2_, promoting the subsequent permeation of ammonium salts into the PbI_2_ framework to form stable perovskite. With this method, they achieved more uniform and smoother perovskite film with large grain size. Gao et al. developed a pre-embedding mixed A-cation halide strategy to transform the residual PbI_2_ near the buried interface into stable 3D perovskite [23]. Moreover, they indicated that this strategy effectively balances lattice strain in the perovskite layer adjacent to the buried interface, contributing to high-efficiency PSCs with superior stability. Nevertheless, Qu et al. demonstrated that residual PbI_2_ clusters are predominantly distributed at the top surface of the TSP perovskite film, forming a phase heterogeneity that disrupting vertical charge transport equilibrium [24]. They further established that achieving phase homogeneity in the TSP perovskite film is essential for high device performance. However, the study did not fully clarify the detrimental effects of PbI_2_ induced phase heterogeneity, particularly in terms of how PbI_2_ cluster-mediated interfacial energy-level mismatches impact the charge transport at the NiO_x_/perovskite interface. Besides, a comprehensive understanding of the unique poor performance of NiO_x_-based TSP p-i-n PSCs remains crucial yet has rarely been documented.

Herein, we elucidate that residual PbI_2_ clusters exhibit vertically gradient distribution across TSP perovskites and mainly accumulate at the top surface of the film. This spatial distribution establishes a Schottky-type heterojunction with FAPbI_3_ perovskite, which induces upward band bending within the perovskite, resulting in substantial energy-level mismatches at both NiO_x_/perovskite and perovskite/C60 interfaces, thus ultimately degrading device performance. To mitigate this problem, we developed a vertical interfacial engineering strategy enabled by the synergistic dual-interface modification of tin trifluoromethanesulfonate (Sn(OTF)_2_) and 4-fluorophenylethylamine chloride (F-PEA), successfully fabricating high-performance NiO_x_-based TSP p-i-n PSCs. Specifically, Sn(OTF)_2_ was employed as a multifunctional buried interface regulator. The functional –OTF groups demonstrate strong coordination bonds with both NiO_x_ and residual PbI_2_, which not only increases the Ni^≥3+^/Ni^2+^ ratio to enhance NiO_x_ conductivity but also converts dense PbI_2_ into a macroporous structure that facilitates ammonium salt penetration, enabling the fabrication of large grain, highly crystalline perovskite films. Moreover, divalent Sn^2+^ ions readily incorporate into the perovskite lattice, generating a Pb–Sn mixed perovskite interlayer at the NiO_x_/perovskite interface. This interlayer effectively elevates the valence band maximum (VBM) of the underlying perovskite from − 5.84 to − 5.53 eV, achieving precise optimization of the NiO_x_/perovskite interfacial energy-level alignment. Complementing the buried interface regulation by Sn(OTF)_2_, F-PEA targets the top surface residual PbI_2_ clusters. It reacts with surface PbI_2_ to form a 2D perovskite layer, which not only passivates bulk and surface defects but also modulates the energy-level alignment at the perovskite/C60 interface, directly resolving the interfacial mismatch caused by surface PbI_2_. Consequently, owing to the synergistic effects of Sn(OTF)_2_ and F-PEA modification, the resulting NiO_x_-based TSP p-i-n PSCs delivered a champion power conversion efficiency (PCE) up to 25.6%, and the unencapsulated devices exhibited exceptional operational stability with a T_80_ time exceeding 1000 h in a N_2_ atmosphere.

## Experimental and Calculation

### Materials

The ITO substrates, nickel oxide (NiO_x_), C60 (99.9%), formamidinium iodide (FAI), and methylammonium iodide (MAI), were purchased from Advanced Election Technology Co., Ltd. The tin trifluoromethanesulfonate (Sn(OTF)_2_) and BCP were purchased from Alfa Aesar. 4-Fluorophenylethylamine chloride (F-PEA), methylammonium chloride (MACl), and lead iodide (PbI_2_) were purchased from Xi’an Yuri Solar Co., Ltd. Dimethyl sulfoxide (DMSO, 99.9%), dimethylformamide (DMF, 99.8%), and isopropanol (IPA, anhydrous, 99.8%) were purchased from Sigma-Aldrich.

### Device Fabrication

ITO glass substrates (7 Ω sq^−1^) were thoroughly cleaned and treated with plasma for 5 min before usage. A layer of NiO_x_ (10 mg mL^−1^ in DI water, filtered through a 0.22-μm PTFE syringe filter) was spin coated at 4000 rpm for 30 s without aging, then annealed at 120 °C for 10 min under ambient conditions. After cooling to room temperature, the substrates were transferred to a nitrogen-filled glovebox. The Sn(OTF)_2_ interlayer was spin coated from the stock solutions (1 mg mL^−1^ in Ethanol) at 3000 rpm for 30 s and annealed at 100 °C for 10 min. Subsequently, a PbI_2_ (1.5 M, DMF/DMSO = 9:1) layer was formed on the substrates using spin coating at 2000 rpm for 30 s, annealing at 70 °C for 60 s. After the PbI_2_ film was cooled to room temperature, FAI/MAI/MACl (90 mg:6.39 mg:9 mg in 1 mL IPA) was spin coated at 2000 rpm for 30 s, followed by thermal annealing at 150 °C for 15 min in an ambient air self-made glovebox with a relative humidity of 10%. For the F-PEA post-treatment, 2 mg mL^−1^ F-PEA in IPA/DMSO = 95:5 solution was spin coated onto the perovskite surface, followed by thermal annealing at 100 °C for 5 min in a nitrogen-filled glovebox. Finally, the samples were transferred to an evaporation chamber where 30 nm C60 at 0.2 Å s^−1^, 8 nm BCP at 0.2 Å s^−1^, and 100 nm Ag at 1.0 Å s^−1^ were deposited under vacuum. The active area of the devices was 0.04 cm^2^.

### Characterizations

All samples were well kept in vacuum-sealed bags during sample transfer. The grazing incidence X-ray diffraction (GIXRD) measurements were conducted on a multifunctional X-ray diffractometer (XPert Pro MPD). Depth-dependent phase information including surface, bulk, and buried interface phases within the perovskite layer can be acquired by adjusting the incident angle (θ) between the X-ray beam and the sample surface. The depth-profiling ultraviolet photoelectron spectroscopy (UPS, ESCALAB 250Xi, Thermo Fisher) measurements were carried out using a He I discharge lamp (21.22 eV). The different etching depths were achieved by a laser marking system. X-ray photoelectron spectroscopy (XPS) was conducted on a Thermo Scientific™ K-Alpha™^+^ spectrometer equipped with a monochromatic Al Kα X-ray source (1486.6 eV) operating at 100 W. All peaks were calibrated with C 1* s* peak binding energy at 284.8 eV for adventitious carbon. For the depth-profiling XPS analysis, the spectra were collected after different Ar-sputtering etching time. The XRD patterns (2θ scans) were obtained on Bruker Advanced D8 X-ray diffractometer using Cu Kα (λ = 0.154 nm) radiation. The ^19^F nuclear magnetic resonance (NMR) spectra were recorded in deuterated dimethyl sulfoxide (DMSO-d6) using a 300 MHz Bruker spectrometer. A UV–Vis spectrophotometer (Agilent Cary 5000) was used to collect the transmittance spectra of the substrates and absorbance spectra of the perovskites. Steady-state photoluminescence (PL) spectra were recorded on Shimadzu RF-5301pc. Time-resolved photoluminescence spectra were measured on a PL system (Fluo-Time 300) under excitation with a picosecond pulsed diode laser at 640 nm wavelength with a repetition frequency of 1 MHz films. The morphology of the films was studied by field-emission scanning electron microscopy (FESEM, TESCAN, MIRA3) and atomic force microscopy (AFM, Bruker Dimension Icon). Current density–voltage (J-V) curves of the devices were collected using a source meter (Keysight B2901A) and a solar simulator (Enlitech SS-F5-3A) with a protocol of 1.2 to − 0.1 V with a 20 mV voltage step and 10 ms delay. The light intensity was calibrated to AM 1.5G (100 mW cm^−2^) by using a reference Si solar cell. The external quantum efficiency (EQE) spectra were recorded with a quantum efficiency measurement system (Enlitech QER-3011) in which the light intensity at every wavelength was calibrated with a Si detector before measurement. The maximum power point (MPP) output was measured by testing the steady-state current density at the maximum power point voltage. Electron only devices with a configuration of ITO/SnO_2_/Perovskite/PCBM/Ag were fabricated for SCLC measurement. The J-V responses of the electron-only devices were measured at RT in the dark. Mott–Schottky analyses were conducted using an electrochemical workstation (IM6eX, Zahner, Germany) under dark conditions. Measurements spanned a voltage range of 0 to 1.5 V, with capacitance–voltage (C–V) curves acquired at a fixed frequency of 5 kHz and a small AC perturbation amplitude of 10 mV. The elemental distribution in perovskite film was characterized using PHI nanoTOF II Time-of-Flight SIMs.

### Density Functional Theory Calculations

First-principles calculations were carried out using the Vienna Ab-initio Simulation Package (VASP), employing the projector augmented wave (PAW) method. The Perdew–Burke–Ernzerhof (PBE) functional within the generalized gradient approximation (GGA) was adopted to describe electron exchange and correlation effects. For the calculations of adsorption energy and defect passivation, a plane-wave cutoff energy of 400 eV was employed, with energy and force convergence criteria set to 1 × 10^–4^ eV and 0.05 eV Å^−1^, respectively.

The NiO_X_ (100) surface was modeled using a 3 × 3 × 1 supercell incorporating a vacuum layer of 15 Å, while the FAPbI_3_ (100) surface was constructed with a 3 × 3 × 2 supercell. A Γ-centered 3 × 3 × 1 Monkhorst–Pack k-point mesh was adopted for the doped NiO_x_ systems, and 1 × 1 × 1 for the doped FAPbI_3_ system. The van der Waals interactions were accounted for using the Grimme DFT-D3 with the zero-damping function in adsorption energy calculations. Additionally, the electrostatic potential (ESP) of the -OTF group was computed using all-electron double-ζ valence basis sets. All crystal structures were constructed using Materials Studio and visualized with VESTA.

## Results and Discussion

### Effects of Vertical Interfacial Engineering on Device Performance

**Fig. 1:**
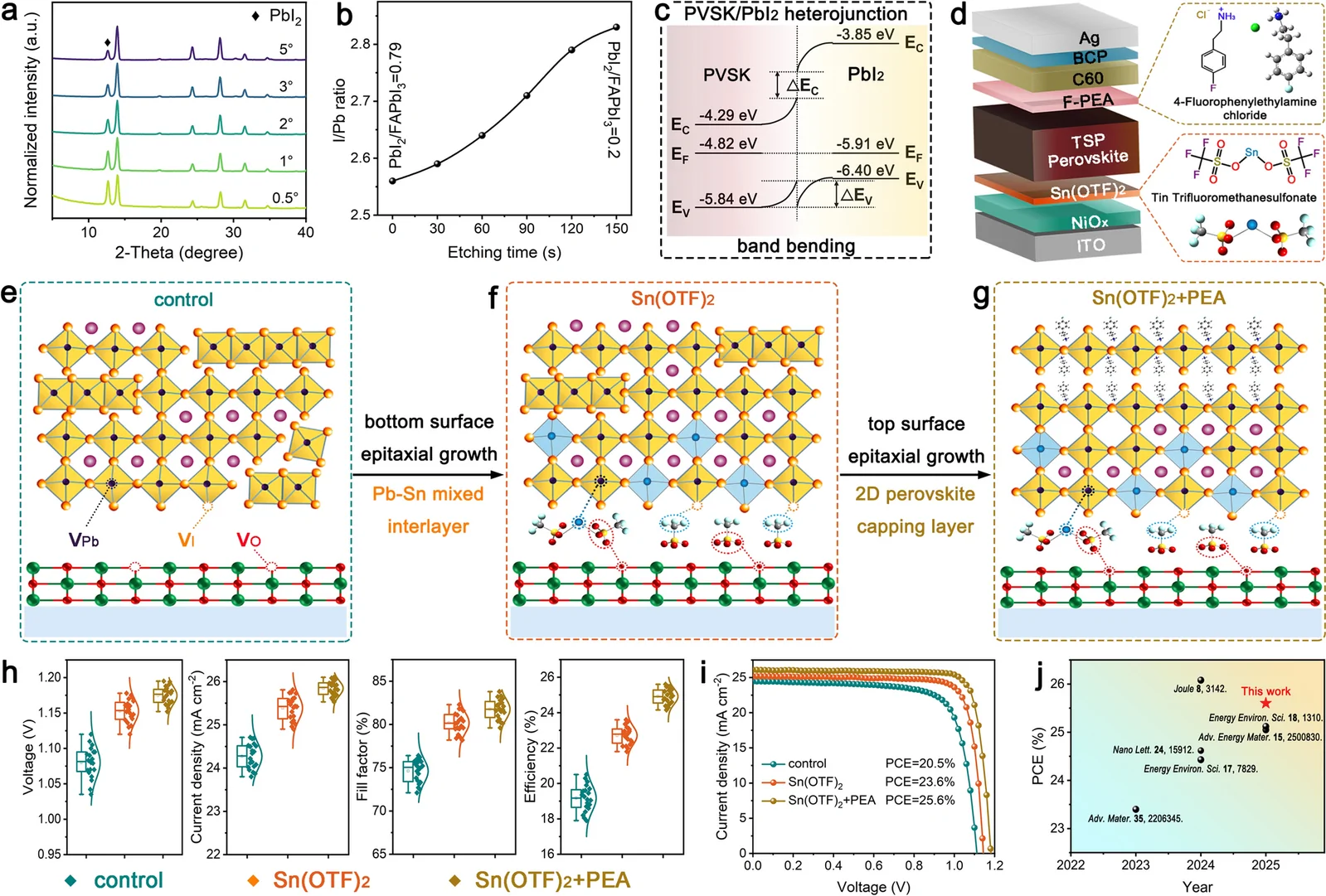
**a** GIXRD patterns of the TSP films with varying incident angles. **b** I/Pb ratio of the TSP film with varying etching time. **c** Schottky junction between PbI_2_ and perovskite. **d** Schematic structure of the NiO_x_-based TSP p-i-n PSCs, and chemical structure of the Sn(OTF)_2_ and F-PEA. Crystallization process of **e** control, **f** Sn(OTF)_2_, and **g** Sn(OTF)_2_ + PEA perovskites. **h** Statistical photovoltaic parameters for different PSCs. A total of 20 devices in 3 batches for each sample were counted. **i** J-V curves of the champion devices based on the control, Sn(OTF)_2_, and Sn(OTF)_2_ + PEA films. **j** Efficiency progress of TSP p-i-n structured PSCs over recent years

SnO_2_-based two-step-processed (TSP) regular n-i-p PSCs have demonstrated high efficiency and reproducibility [25–27]. However, while demonstrating potential for tandem applications, NiO_x_-based TSP p-i-n PSCs show significantly inferior photovoltaic performance (Fig. S1) and have been rarely documented. To delve into the underlying mechanisms governing this performance disparity, the phase homogeneity in TSP FAPbI_3_ perovskite film was investigated using grazing incidence X-ray diffraction (GIXRD) with varying incident angles from 0.5° to 5°. As shown in Fig. 1a, the TSP film exhibits distinct diffraction peaks at 12.8°, which correspond to PbI_2_, indicating the existence of residual PbI_2_ within the film. Additionally, the diffraction intensity of the PbI_2_-related peaks gradually decreases with increasing incident angles. This observation suggests that the residual PbI_2_ is predominantly distributed near the top surface of the film. To quantitatively evaluate the distribution of PbI_2_, the perovskite film was exfoliated from the substrate (Fig. S2), and X-ray photoelectron spectroscopy (XPS) was conducted to analyze the chemical states at the top and bottom surfaces. As shown in Fig. S3, the bottom surface of the TSP perovskite film exhibits significantly stronger methylammonium (MA^+^) and formamidinium (FA^+^) cation signals compared to the top surface, corroborating the conclusion that residual PbI_2_ is predominantly distributed near the top surface. This is further supported by the Pb 4*f* core-level spectra, which exhibit a lower binding energy shift at the bottom surface, consistent with reduced PbI_2_ content as previously reported [24]. Furthermore, depth-profiling XPS was performed to determine the I/Pb ratio across different etching times (Fig. S4 and Table S1). This ratio primarily reflects contributions from FAPbI_3_ perovskite and residual PbI_2_ clusters. As shown in Fig. 1b, increasing etching time raised the I/Pb ratio from 2.56 (0 s) to 2.83 (150 s), which lowered the PbI_2_/FAPbI_3_ ratio from 0.79 to 0.2. These results indicate a higher proportion of FAPbI_3_ and less PbI_2_ with increasing etching time, further confirming that residual PbI_2_ is mainly distributed near the top surface of the TSP film, consistent with previous studies. As illustrated in Fig. 1c, the residual PbI_2_, as a wide-bandgap semiconductor, tends to form a Schottky contact with FAPbI_3_, resulting in a band bending in the perovskite [28]. Consequently, the inhomogeneous PbI_2_ distribution disrupts the vertical energy-level alignment in perovskites, thereby impairing charge carrier transport and device efficiency [29].

To this end, a vertical interfacial engineering enabled by dual-interface modification was developed to fabricate high-performance NiO_x_-based TSP p-i-n PSCs. As depicted in Fig. 1d, functional tin trifluoromethanesulfonate (Sn(OTF)_2_) and 4-Fluorophenylethylamine chloride (F-PEA) were spin coated onto NiO_x_ and perovskite surface, respectively, as interfacial layers during device fabrication. Their working mechanisms are schematically illustrated in Fig. 1e-g. Depositing pristine TSP films on bare NiO_x_ (denoted as the control) introduces abundant defects at the NiO_x_/perovskite interface, including oxygen vacancies (V_O_), undercoordinated Ni^≥3+^ species, iodine vacancies (V_I_), and Pb vacancies (V_Pb_). These defects facilitate adverse redox reactions, accelerate ion migration from the perovskite layer to NiO_x_, and ultimately degrade device performance (Fig. 1e) [30]. Besides, the compact pre-deposited PbI_2_ film hinders ammonium salt penetration, leaving control films with numerous heterogeneously distributed residual PbI_2_ clusters [31]. Intriguingly, with the introduction of the Sn(OTF)_2_ interlayer (referred to as Sn(OTF)_2_ hereafter), the aforementioned adverse redox reactions and ions migration are effectively suppressed via the passivation effect originated from the –OTF groups. Moreover, the divalent Sn^2+^ ions in Sn(OTF)_2_ can partially compensate for V_Pb_ through incorporation into the perovskite lattice (Fig. 1f), forming a Pb–Sn mixed perovskite interlayer which is critical for energy-level alignment at the buried NiO_x_/perovskite interface. Simultaneously, the trifluoromethanesulfonate –OTF groups exhibit strong coordination with PbI_2_ through –SO_3_^−^…Pb ionic bonding and − CF_3_…Pb hydrogen bonding, as evidenced by the ^19^F NMR spectra (Fig. S5) [32]. This interaction slows PbI_2_ crystallization and yields a uniform macroporous PbI_2_ framework as evidenced by the XRD amd SEM results (Fig. S6). This enhances ammonium salt penetration and reduces residual PbI_2_ clusters at the interface. Finally, F-PEA post-treatment induces the formation of 2D perovskite capping layer at the top surface of the perovskite (referred to as Sn(OTF)_2_ + PEA hereafter, Fig. 1g) [33]. The formation of this 2D perovskite not only reduces residual PbI_2_ clusters near the top surface of the film but also passivates defects and optimizes energy-level alignment at the perovskite/C60 interface. Building on these advantages, it is expected that the vertical interfacial engineering can significantly enhance the efficiency and stability of the resulting NiO_x_-based TSP p-i-n PSCs.

To evaluate the efficacy of the Sn(OTF)_2_ interlayer and F-PEA post-treatment on device performance, NiO_x_-based TSP p-i-n PSCs (Fig. 1d) were fabricated. The corresponding cross-sectional SEM image of the device exhibits a uniform stacking of functional layers (Fig. S7). Figure 1h summarizes statistical photovoltaic parameters of the devices based on the control, Sn(OTF)_2_, and Sn(OTF)_2_ + PEA films. It is found that the Sn(OTF)_2_ + PEA devices demonstrate an overall enhancement on open-circuit voltage (V_oc_), short-circuit current density (J_sc_), and fill factor (FF), resulting in the best PCEs. Figures 1i and S8a–c show the photocurrent density–voltage (J-V) curves of the champion devices, corresponding photovoltaic parameters are summarized in Table S2. Hysteresis analysis based on H-index (HI) reveals that the Sn(OTF)_2_ + PEA device has the smallest HI value (1.9%) compared to the control (9.3%) and Sn(OTF)_2_ (4.7%) devices. The external quantum efficiency (EQE) integrated J_sc_ is 23.51, 24.34, and 25.16 mA cm^−2^ for the control, Sn(OTF)_2_, and Sn(OTF)_2_ + PEA devices, respectively, which is well matched with the J_sc_ extracted from the J-V curves (Fig. S8d). To confirm the reliability of the J-V measurements, steady-state power output (SPO) at the maximum power point was recorded (Fig. S8e). The PCE of the control, Sn(OTF)_2_, and Sn(OTF)_2_ + PEA devices stabilized at 20.1%, 23.2%, and 25.1%, respectively, which are consistent with the J-V results. Besides, it should be mentioned that the Sn(OTF)_2_ + PEA device gave a champion PCE of 25.6% under reverse scan with a V_oc_ of 1.18 V, a J_sc_ of 26.1 mA cm^−2^, an FF of 82.8%. This PCE represents one of the highest efficiencies recorded for TSP p-i-n structured PSCs thus far (Fig. 1j).

### Influence of Sn(OTF)_2_ on the Properties of NiO_x_

**Fig. 2:**
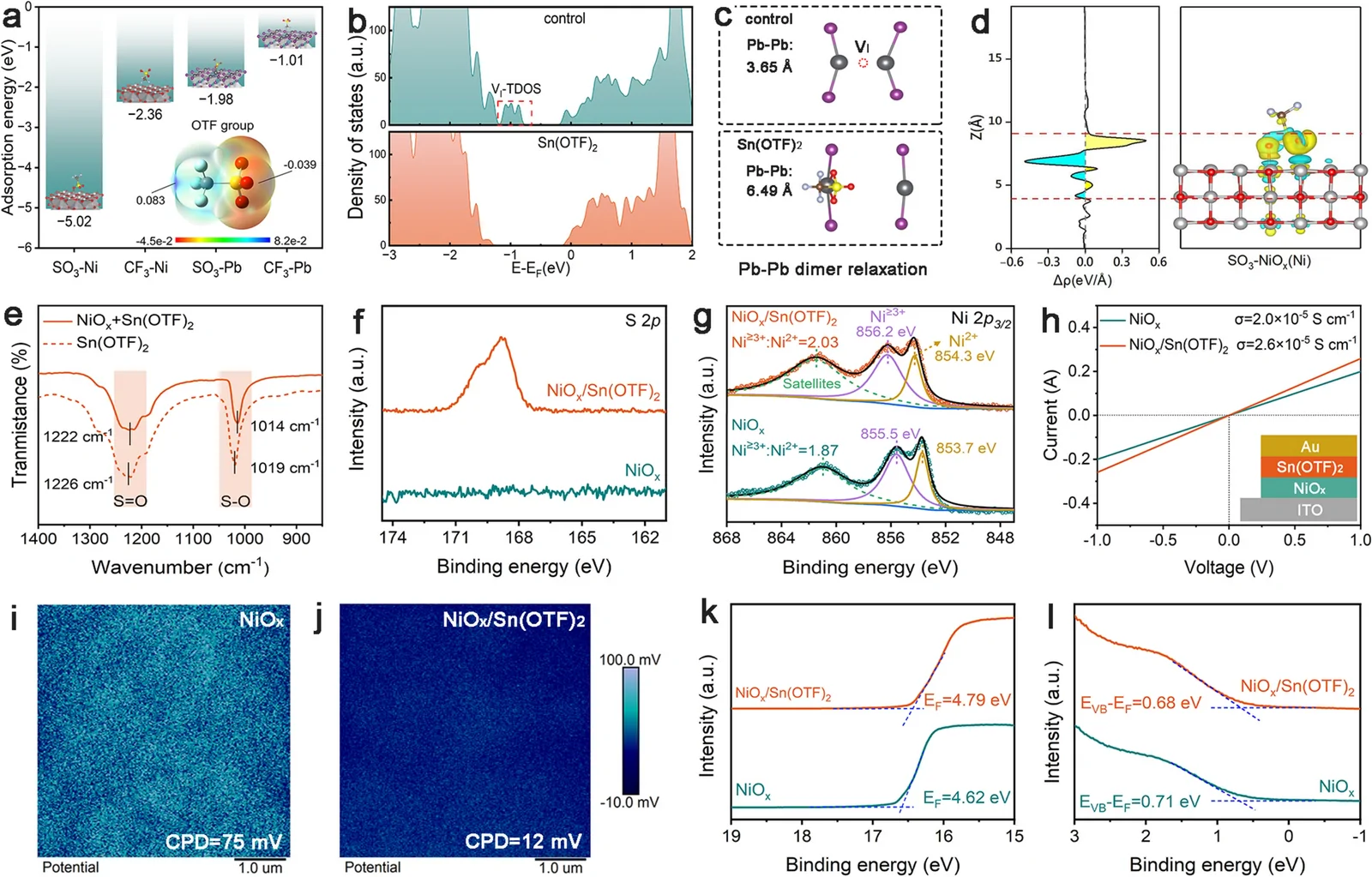
**a** Adsorption energies of − SO_3_^−^ and − CF_3_ groups with NiO_x_ and perovskite, and the electrostatic potential profile of the –OTF group. **b** The density of states of the surface electron-injected V_I_ defect for the control and Sn(OTF)_2_ films. **c** Schematic diagram of an iodine vacancy with trapping an electron to form a Pb − Pb dimer and then passivated by Sn(OTF)_2_. **d** Differential charge density mapping depicting the interaction of the − SO_3_^−^ group with the NiO_x_ layer. **e** FTIR spectra of pure Sn(OTF)_2_ and NiO_x_ + Sn(OTF)_2_. XPS core spectra of **f** S 2*p* and **g** Ni 2*p*_3/2_ for bare NiO_x_ and NiO_x_/Sn(OTF)_2_ films. **h** I-V characteristics of the different films for evaluating the corresponding conductivity. KPFM images of **i** bare NiO_x_ and **j** NiO_x_/Sn(OTF)_2_ films. UPS spectra of secondary electron **k** cutoff (E_cutoff_) and **l** onset (E_onset_) energy

To elucidate the dual-anchoring capability of Sn(OTF)_2_ at the NiO_x_/perovskite interface and its passivation effects on the perovskite layer, density functional theory (DFT) calculations were conducted using the Vienna Ab-initio Simulation Package (VASP). The calculations specifically focus on the functional –OTF groups. As shown in the inset of Fig. 2a, the electrostatic potential surface (EPS) of the –OTF group reveals a strong electron-withdrawing region (red, negative potential) localized around the –SO_3_^−^ group, while the electron-donating region (blue, positive potential) is primarily localized near the –CF_3_ end. The oxygen atoms in the –SO_3_^−^ group can effectively coordinate with undercoordinated Ni^3+^ or Pb^2+^ ions at the NiO_x_/perovskite interface, thereby reducing defect-induced trap states. To evaluate the anchoring preference of the –OTF group, adsorption energies (E_ad_) were calculated for both terminal groups (–SO_3_^−^ and –CF_3_) on NiO_x_ (001) and perovskite (001) surfaces (Fig. S9). The –SO_3_^−^ group demonstrates a substantially lower E_ad_ value on NiO_x_ (− 5.02 eV) compared to the –CF_3_ group (− 2.36 eV), indicating a significantly stronger binding affinity toward the NiO_x_ surface. Conversely, both moieties displayed weaker binding on the perovskite (001) surface, with E_ad_ values of − 1.98 eV for –SO_3_^−^ and − 1.01 eV for –CF_3_. These computational results demonstrate that –OTF groups exhibit two distinct adsorption modes at the NiO_x_ surface. The majority of –SO_3_^−^ moieties preferentially anchor to the NiO_x_ surface, while –CF_3_ interact specifically with undercoordinated Pb^2+^ sites in adjacent perovskite layers, establishing dual-anchoring behavior. However, in regions with either incomplete NiO_x_ coverage or excessive—OTF loading (over-coverage), residual unbound—SO_3_^−^ groups may interact with the perovskite surfaces through ionic bonding, enhancing interfacial connectivity while optimizing structural configuration.

It has been reported that under continuous illumination or applied electric fields, iodine vacancies (V_I_) readily form on perovskite surfaces [34]. These vacancies trap excess electrons and facilitate Pb-Pb dimer formation via attractive interactions between adjacent Pb atoms, thereby compromising structural stability [35]. As shown in Fig. 2b, these dimers introduce deep-level trap states within the bandgap, enhancing non-radiative recombination and decreasing carrier lifetimes [36]. The –OTF groups mitigate this issue through interaction with Pb^2+^, effectively passivating trap states and restoring a clean bandgap. DFT results demonstrate a substantial increase in Pb–Pb interatomic distance from 3.65 to 6.49 Å following –OTF passivation (Fig. 2c), providing direct evidence of dimer dissociation. Additionally, owing to the strong affinity of the –SO_3_^−^ group for NiO_x_, the differential charge density (Δρ) at the –OTF/NiO_x_ interface was analyzed. Figure 2d demonstrates that –SO_3_^−^ group interacts with NiO_x_ through S = O⋯Ni coordination bonds, resulting in electron depletion on NiO_x_ and accumulation on –OTF. This charge redistribution facilitates hole transfer to NiO_x_, thereby improving interfacial charge extraction efficiency.

Fourier-transform infrared (FTIR) spectroscopy was adopted to experimentally study the above-mentioned interactions. As presented in Fig. 2e, the S = O and S–O stretching vibrations of pure Sn(OTF)_2_ exhibited red shifts from 1226 to 1222 cm^−1^ and 1019 to 1014 cm^−1^, respectively, in the NiO_x_ + Sn(OTF)_2_ sample. This lower wavenumber shifted S = O and S–O peaks validate the interaction between NiO_x_ and the –SO_3_^−^ group. Besides, XPS measurement was conducted to analyze the chemical states of the deposited NiO_x_ and NiO_x_/Sn(OTF)_2_ thin films. The detection of a distinct S 2*p* XPS peak (Fig. 2f) confirms the successful introduction of Sn(OTF)_2_ onto the NiO_x_ surface. Figure 2g shows the Ni 2*p*_3/2_ core spectra of the films, which were deconvoluted into three peaks corresponding to satellite states, Ni^≥3+^ and Ni^2+^ species [37]. Notably, the Ni 2*p*_3/2_ core spectra of the NiO_x_/Sn(OTF)_2_ film exhibit a positive binding energy shift, corroborating the interaction between NiO_x_ and Sn(OTF)_2_. Furthermore, as determined by the integral areas of the respective peaks, the Ni^≥3+^/Ni^2+^ ratio increased from 1.87 to 2.03 for the NiO_x_ and NiO_x_/Sn(OTF)_2_ films, respectively, which can be attributed to electron transfer from Ni to electron-withdrawing –SO_3_^−^ groups. In general, higher Ni^≥3+^ content in NiO_x_ films correlates with improved electrical conductivity [12]. To quantify this, current–voltage (I-V) measurements were conducted on devices with the architecture illustrated in the inset of Fig. 2h, with detailed calculations and parameters summarized in Table S3. Accordingly, the NiO_x_/Sn(OTF)_2_ film demonstrates significantly enhanced conductivity (2.6 × 10^–5^ S cm^−1^) compared to bare NiO_x_ (2.0 × 10^–5^ S cm^−1^), which is beneficial for hole transport.

Subsequently, the surface properties of NiO_x_ and NiO_x_/Sn(OTF)_2_ films were systematically investigated. Figure S10 presents the surface morphology and roughness of the films. The scanning electron microscopy (SEM) images indicate that Sn(OTF)_2_ does not alter the morphology of NiO_x_ films. In contrast, atomic force microscopy (AFM) results demonstrate a reduction in surface roughness, with root-mean-square (RMS) roughness decreasing from 5.29 ± 0.34 nm (bare NiO_x_) to 4.76 ± 0.18 nm (NiO_x_/Sn(OTF)_2_). This roughness reduction confirms the formation of a continuous and pinhole-free Sn(OTF)_2_ interlayer, which is a prerequisite for its passivation and coordination functions. Furthermore, the reduced RMS roughness eliminates localized peaks/valleys, ensuring uniform coverage of the perovskite layer on the NiO_x_ surface and maximal contact with the Sn(OTF)_2_ interlayer, which is critical for charge carrier extraction [38]. Further, Kelvin probe microscopy (KPFM) measurements were conducted to evaluate the surface potential of the films. As shown in Fig. 2i, j, contact potential difference (CPD) values for NiO_x_ and NiO_x_/Sn(OTF)_2_ films were 75 and 12 mV, respectively. The lower CPD value in NiO_x_/Sn(OTF)_2_ film indicates a larger work function compared to the bare NiO_x_ [39, 40]. To quantitatively assess film work functions, ultraviolet photoelectron spectroscopy (UPS) measurements were performed. Figure 2k presents the secondary electron cutoff (E_cutoff_) energies of the NiO_x_ and NiO_x_/Sn(OTF)_2_ films, from which the corresponding Fermi level (E_F_) values were determined to be − 4.62 and − 4.79 eV, respectively. The downward-shifted E_F_ in NiO_x_/Sn(OTF)_2_ film confirms the increased work function, which is consistent with the KPFM results. Figure 2l shows the energy gap between the VBM and E_F_ of the films. A smaller gap (0.68 eV) in NiO_x_/Sn(OTF)_2_ film indicates stronger p-type doping, correlating with the higher Ni^≥3+^/Ni^2+^ ratio observed via XPS. Consequently, the NiO_x_/Sn(OTF)_2_ film demonstrated a deeper VBM (− 5.47 eV) than that of bare NiO_x_ film (-5.33 eV, Table S4). This energy-level adjustment is essential for mitigating the energy-level mismatch at the NiO_x_/perovskite interface. Additionally, Fig. S11 presents the optical transmission spectra of films deposited on Glass/indium tin oxide (ITO) substrates. Both NiO_x_ and NiO_x_/Sn(OTF)_2_ films exhibit high transmittance across the visible spectrum, which is favorable for highly efficient PSCs fabrication.

### Effects of Dual-Interface Modification on Perovskite Crystallization and Properties

**Fig. 3:**
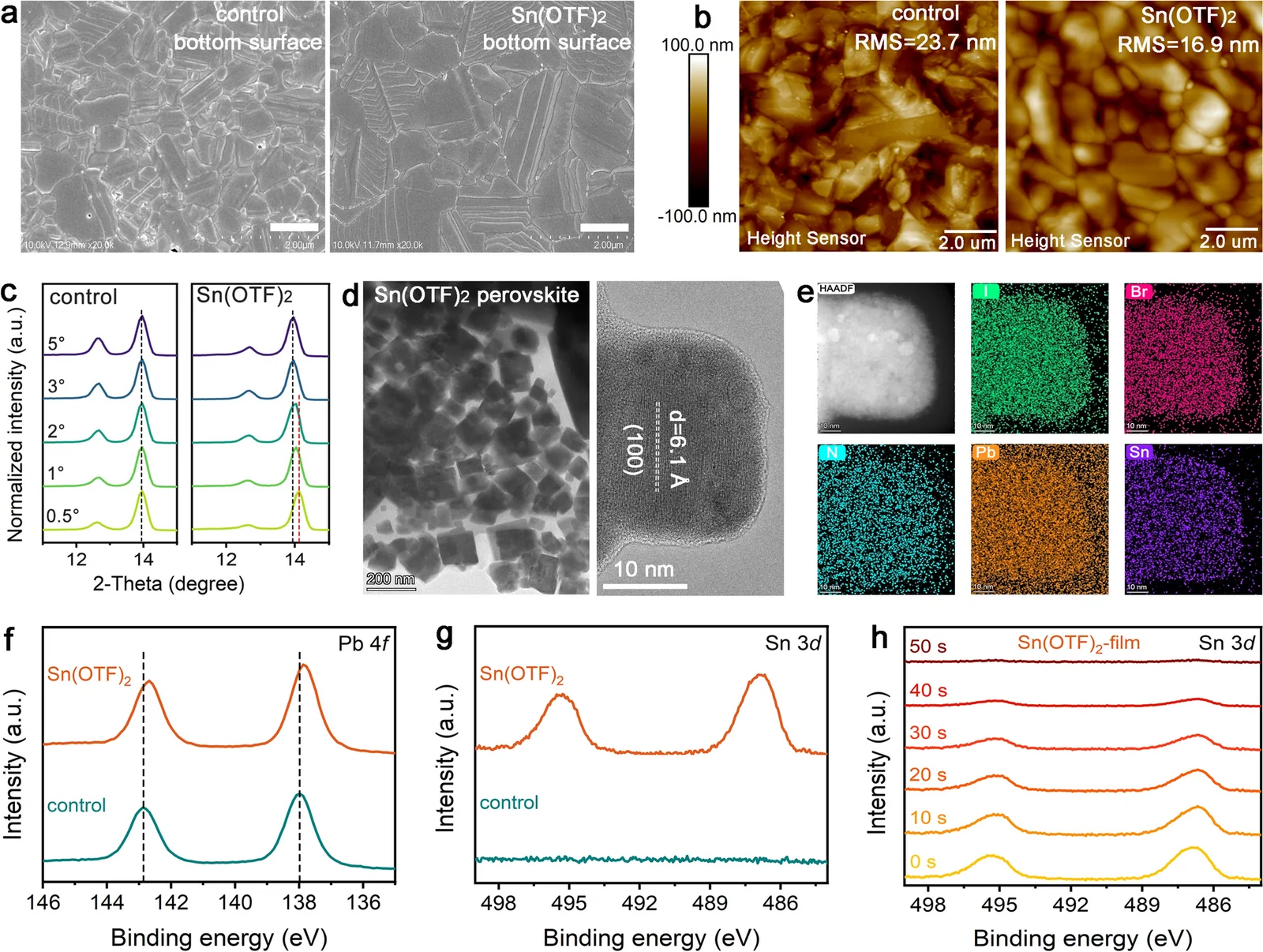
Bottom-view **a** SEM images and **b** AFM images of the control and Sn(OTF)_2_ films. **c** Enlarged GIXRD spectra of the control and Sn(OTF)_2_ films acquired from the bottom surface. **d** TEM image and **e** EDXS mapping of key elements in HAADF mode for Sn(OTF)_2_ perovskite crystal. **f** Pb 4*f* and **g** Sn 3*d* core spectra of the control and Sn(OTF)_2_ films. **h** Depth-profiled Sn 3*d* core spectra of the Sn(OTF)_2_ film with varying etching time

Due to the direct contact with Sn(OTF)_2_ interlayer, the crystallization and quality of the perovskite should be affected [41]. To delve into the effect of the Sn(OTF)_2_ interlayer on TSP perovskite crystallization, in situ photoluminescence (PL) spectroscopy was conducted. As shown in Fig. S12, the perovskite PL peak (λ = 800 nm) for the control film emerges within 5 s of annealing, and the PL intensity saturates at approximately 20 s, indicating complete crystallization. This rapid rate corresponds to the abundant nucleation sites in dense PbI_2_, resulting in swift perovskite formation. In contrast, for the Sn(OTF)_2_ film, an initial PL peak at λ = 850 nm (5–10 s) corresponds to early-stage Pb–Sn perovskite, indicating the incorporation of Sn^2+^ from Sn(OTF)_2_ into the lattice. Subsequently, the peak shifts to λ = 800 nm as the organic salts (FAI, MAI, and MACl) fully diffuse and react, signifying mature perovskite formation. The corresponding PL intensity saturates at around 30 s, which is 50% longer than that of the control. This slower rate aligns with the reduced nucleation density of the porous PbI_2_ template, leading to longer grain growth times but larger grain sizes. To validate the enlarged perovskite grains, the perovskite films were exfoliated from the substrates. Figure 3a shows bottom-view SEM images of the control and Sn(OTF)_2_ films. It is noticeable that the control film exhibits a bottom surface morphology with small grains and minor pinholes, whereas the Sn(OTF)_2_ film features a pinhole-free bottom surface morphology with significantly larger grains, which is in accordance with the in situ PL results. Corresponding AFM images (Fig. 3b) of the perovskites suggest that Sn(OTF)_2_ film exhibits a smoother bottom surface (RMS = 16.9 nm), demonstrating improved interfacial contact between the perovskite layer and substrate, which is beneficial for high device performance [42]. The increase in perovskite grain size, decrease in pinhole density, and reduction in RMS roughness observed in the Sn(OTF)_2_ film stems primarily from the –OTF functional groups of Sn(OTF)_2_. These functional groups coordinate with PbI_2_, inducing the formation of a uniform macroporous PbI_2_ film (Fig. S6). This facilitates organic salt diffusion throughout the film and promotes perovskite nucleation and growth of larger grains. Bottom surface GIXRD measurements were conducted to investigate the structural properties of the films. As shown in Fig. S13, the Sn(OTF)_2_ film exhibits a much weaker PbI_2_ peak intensity than the control film at the same incident angles. This observation indicates that the Sn(OTF)_2_ interlayer facilitates the transformation from PbI_2_ to perovskite, resulting in highly crystalline perovskite film with reduced residual PbI_2_ clusters at the bottom. Additionally, the enlarged (100) perovskite planes (Fig. 3c) reveal peak shifts toward higher angles in the Sn(OTF)_2_ film at incident angles lower than 3°. This implies Sn^2+^ ions (with smaller ionic radii) have incorporated into the perovskite lattice, forming a Pb–Sn mixed perovskite phase. As the incident angles increased beyond 3°, no significant peak shift can be observed, suggesting that the Pb–Sn mixed perovskite layer is predominantly distributed near the bottom surface of the Sn(OTF)_2_ perovskite film. To further validate the incorporation of Sn^2+^ ions, high-resolution transmission electron microscopy (HRTEM) analysis was performed (Fig. 3d). It is noteworthy that well-defined lattice fringes of perovskite crystal can be found, indicating excellent crystallinity. Besides, the measured (100) d-spacing of 6.1 Å is relatively smaller than that of pure Pb-based perovskite (6.3 Å), consistent with the substitution of Pb^2+^ (ionic radius 1.19 Å) by smaller Sn^2+^ ions (1.10 Å). Furthermore, high-angle annular dark-field (HAADF) energy-dispersive X-ray spectroscopy (EDXS) elemental mappings of the perovskite crystal (Fig. 3e) demonstrate uniform spatial distributions of I, Br, N, and Pb. Critically, the distinct Sn signal provides direct evidence for successful Sn^2+^ incorporation in the final perovskite film.

Besides, the chemical states at the bottom surface of the perovskites were also investigated. As presented in Fig. 3f, the Pb 4*f* core spectra in the Sn(OTF)_2_ film exhibit a lower binding energy shift compared to the control film. This binding energy shift is mainly attributed to synergistic interactions between –SO_3_^−^ and –CF_3_ groups (from –OTF groups) with PbI_2_, as evidenced by combined ^19^F NMR analysis (Fig. S5) and DFT-calculated adsorption energies (Fig. S9). These interactions effectively passivate V_I_ defects, consistent with the defect passivation mechanism demonstrated in our DFT simulations (Fig. 2b). Moreover, the detection of distinct Sn 3*d* XPS peaks (Fig. 3g) in the Sn(OTF)_2_ film further confirms the successful incorporation of Sn^2+^ ions into the perovskite lattice. The Sn/Pb atomic ratio N_Sn_/N_Pb_ was calculated as 0.11 according to the equation: ${{N_{Sn} } \mathord{\left/ {\vphantom {{N_{Sn} } {N_{Pb} }}} \right. \kern-0pt} {N_{Pb} }} = {{\left( {{{A_{Sn} } \mathord{\left/ {\vphantom {{A_{Sn} } {S_{Sn} }}} \right. \kern-0pt} {S_{Sn} }}} \right)} \mathord{\left/ {\vphantom {{\left( {{{A_{Sn} } \mathord{\left/ {\vphantom {{A_{Sn} } {S_{Sn} }}} \right. \kern-0pt} {S_{Sn} }}} \right)} {\left( {{{A_{Pb} } \mathord{\left/ {\vphantom {{A_{Pb} } {S_{Pb} }}} \right. \kern-0pt} {S_{Pb} }}} \right)}}} \right. \kern-0pt} {\left( {{{A_{Pb} } \mathord{\left/ {\vphantom {{A_{Pb} } {S_{Pb} }}} \right. \kern-0pt} {S_{Pb} }}} \right)}}$, where ${A}_{Sn}$ (50,142) and ${A}_{Pb}$ (775,635) represent the Sn 3*d* and Pb 4*f* peak areas, and ${S}_{Sn}$ (4.095) and ${S}_{Pb}$ (6.968) are their respective sensitivity factors [43]. This indicates that the bottom Pb–Sn mixed perovskite interlayer owns a composition of FASn_0.1_Pb_0.9_I_3_. According to previous studies [44], the VBM of Pb–Sn perovskite is determined by interactions between Sn-*s* and I-*p* orbitals, leading to VBM positions higher than their Pb-only counterparts. Thus, the FASn_0.1_Pb_0.9_I_3_ perovskite interlayer effectively elevates the VBM of the perovskite, which critically optimizing NiO_x_/perovskite interfacial energy-level alignment. Additionally, depth-profiling XPS measurements were performed to determine the thickness of the FASn_0.1_Pb_0.9_I_3_ interlayer. As shown in Fig. 3h, the peak intensity of the Sn 3*d* spectra progressively decreases with increasing etching time and disappears entirely after 50 s of etching. Based on the measured thickness of the perovskite film (700 nm, Fig. S7) and the Pb 4*f* depth profile (Fig. S14), the thickness of the FASn_0.1_Pb_0.9_I_3_ interlayer was calculated to be approximately 106 nm.

**Fig. 4:**
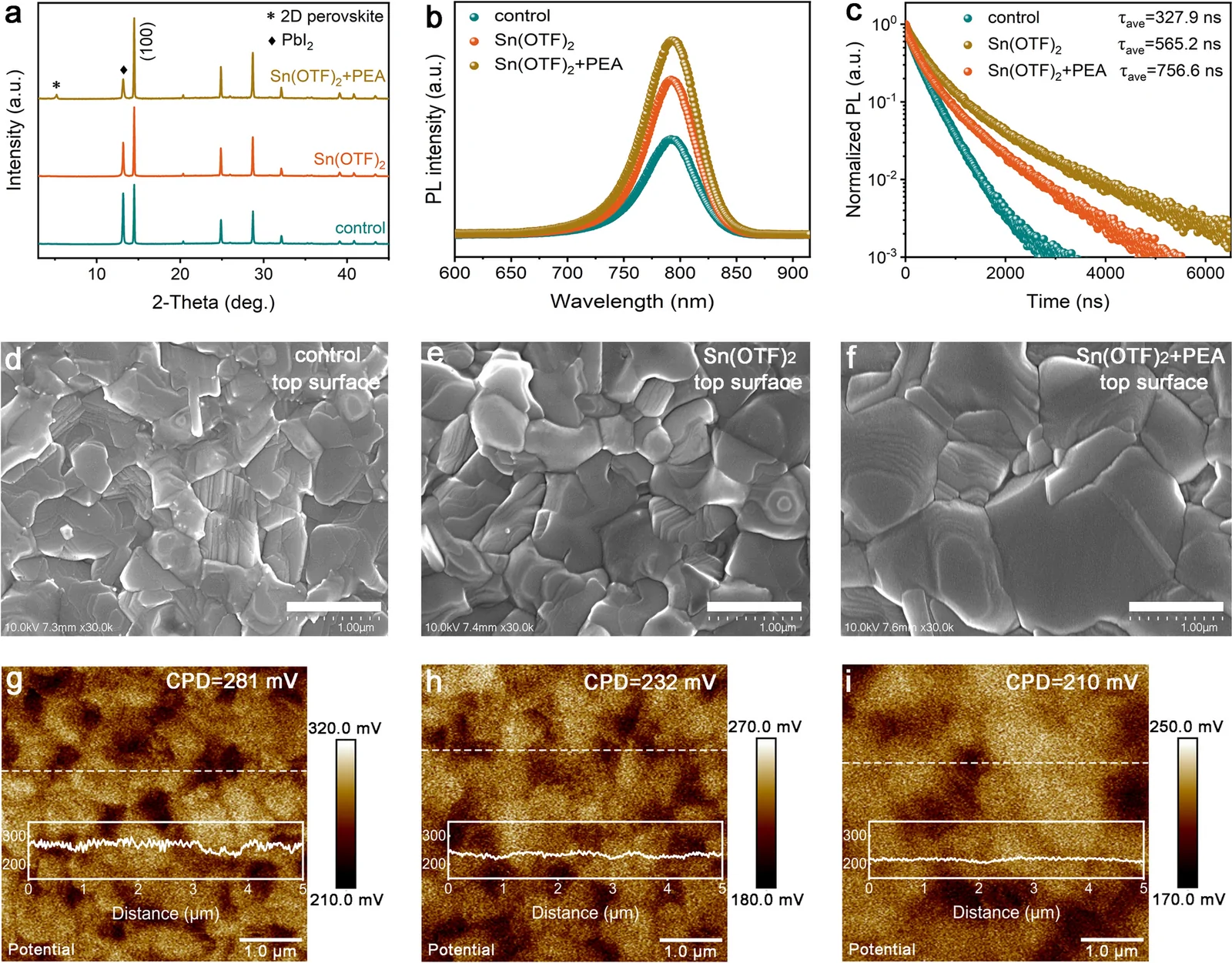
**a** XRD patterns, **b** steady-state PL spectra, and **c** TRPL spectra of the control, Sn(OTF)_2_, and Sn(OTF)_2_ + PEA films. **d-f** Top-view SEM images and **g-i** KPFM images of the different films. The scale bar is 1 μm

To further investigate the effect of Sn(OTF)_2_ interlayer and F-PEA post-treatment on perovskite crystallinity, XRD measurements were conducted. As presented in Fig. 4a, all films show distinct diffraction peaks corresponding to the perovskite phase. The Sn(OTF)_2_ + PEA film displays the strongest (100) perovskite peak and weakest PbI_2_ peak, indicating superior crystallinity and the lowest residual PbI_2_ content. Additionally, a new diffraction peak at around 5.2° in the Sn(OTF)_2_ + PEA film confirms the formation of 2D perovskite, which originating from reactions between F-PEA and residual PbI_2_ clusters at the perovskite top surface [33]. The UV–visible absorption spectra of the perovskite films are presented in Fig. S15. Notably, the Sn(OTF)_2_ + PEA film exhibits the strongest absorption across the visible region, which can be attributed to its superior crystallinity. Additionally, the Urbach tail defects in the films were evaluated, and the Sn(OTF)_2_ + PEA film yielded the smallest Urbach energy (E_u_) of 36.5 meV. This low E_u_ value indicates enhanced crystallinity, which is consistent with the XRD results [45]. Figure 4b displays the steady-state photoluminescence (PL) spectra of the films deposited on quartz glass. The characteristic emission peak is observed at 795 nm, which correlates well with the UV–Vis absorption results. The strongest PL intensity indicates that the non-radiative recombination is substantially suppressed within the Sn(OTF)_2_ + PEA film. Time-resolved PL (TRPL) measurements were performed to evaluate charge carrier lifetimes, and the results were analyzed using a biexponential decay function, with detailed parameters provided in Table S5. As shown in Fig. 4c, the calculated average carrier lifetimes of the control, Sn(OTF)_2_, and Sn(OTF)_2_ + PEA films were 327.9, 565.2, and 756.6 ns, respectively. The longest carrier lifetime in the Sn(OTF)_2_ + PEA film is primarily ascribed to the enhanced crystallinity and suppressed non-radiative recombination [46].

The surface morphologies of the different perovskite films were investigated using top-view SEM and AFM. As presented in Fig. 4d–f, the Sn(OTF)_2_ + PEA film exhibits significantly larger grain sizes (exceeding 2 μm) compared to the control (below 1 μm) and Sn(OTF)_2_ films (approximately 1 μm). This distinct difference in morphology primarily arises from the recrystallization process mediated by F-PEA post-treatment, resulting in the formation of 2D perovskite capping layer, as supported by the XRD results. Figure S16 shows the RMS values of the control, Sn(OTF)_2_, and Sn(OTF)_2_ + PEA films, which are 39.9, 37.2, and 33.7 nm, respectively. The Sn(OTF)_2_ + PEA film exhibits the smallest surface roughness, which is crucial for achieving high device performance. Corresponding KPFM results (Fig. 4g–i) reveal a reduction in CPD values from 281 mV for the control film to 232 mV for the Sn(OTF)_2_ film and 210 mV for the Sn(OTF)_2_ + PEA film. As discussed earlier, a decrease in CPD values correlates with an increase in the work function of the respective films, which facilitates balanced energy-level alignment at the perovskite/C_60_ interface. Additionally, the line profiles of the control and Sn(OTF)_2_ films exhibit significant variations than those of the Sn(OTF)_2_ + PEA film. These variations can be attributed to a high density of defects on the surface of these perovskite film. Thus, the mitigated CPD variation in the Sn(OTF)_2_ + PEA film indicates that F-PEA post-treatment effectively passivates surface defects.

### Energy-Level Alignment and Charge Dynamic Studies

**Fig. 5:**
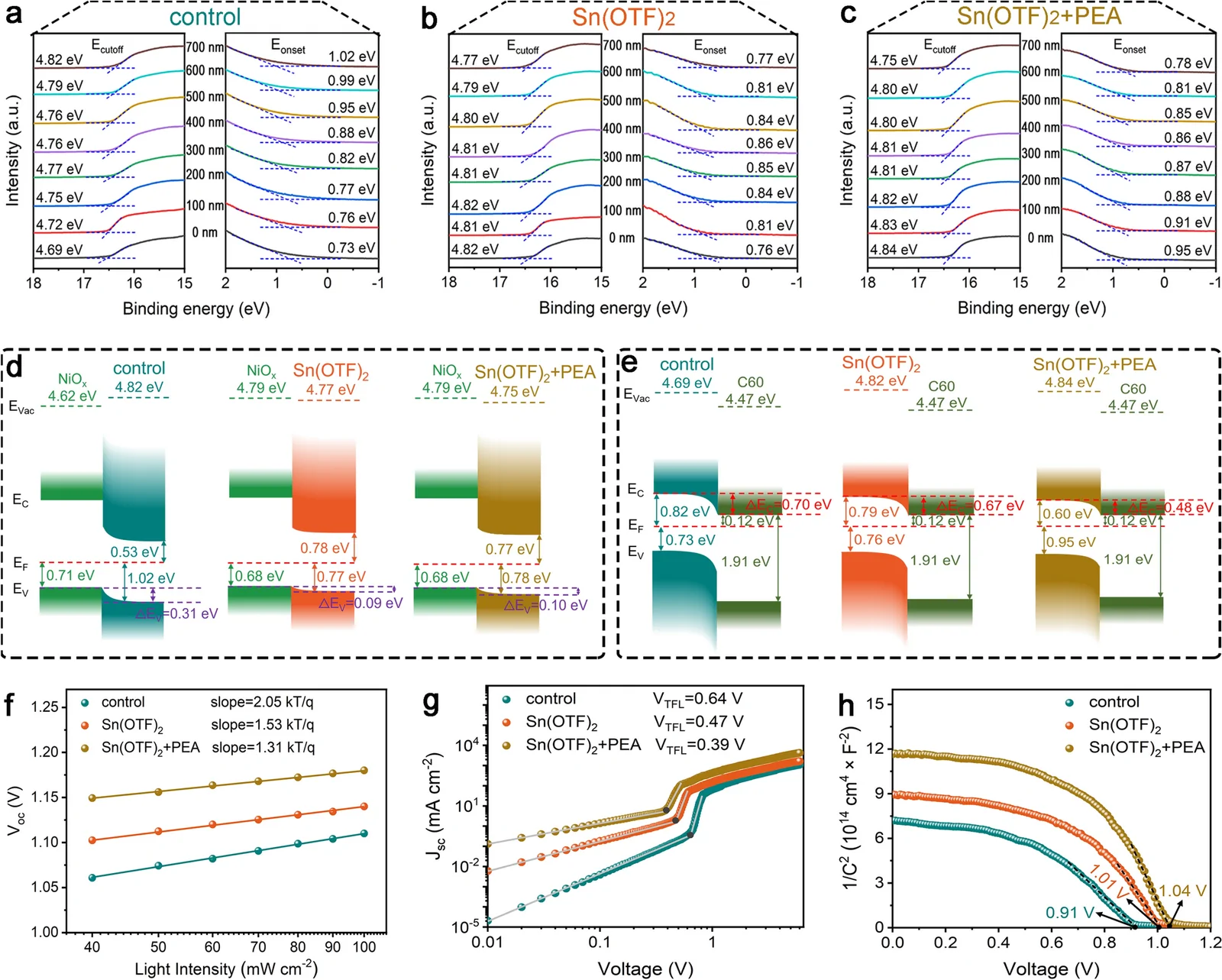
Depth-profiling UPS spectra of of the **a** control, **b** Sn(OTF)_2_, and **c** Sn(OTF)_2_ + PEA films. Energy-level diagrams of the TSP p-i-n PSCs at the **d** NiOx/perovskite and **e** perovskite/C60 interfaces. **f** V_oc_ response under different light intensities of the PSCs. **g** Dark J-V curves for the electron-only devices. **h** Mott–Schottky results for devices based on different films

To comprehensively investigate the effects of the Sn(OTF)_2_ interlayer and F-PEA post-treatment on the energy-level structure of perovskite, depth-profiling UPS measurements were performed to determine the energy-level positions of the control, Sn(OTF)_2_, and Sn(OTF)_2_ + PEA perovskite films at varying etching depths. Figure 5a–c presents the obtained E_cutoff_ and E_onset_ spectra for the different films. By combining with the optical bandgap of pure FAPbI_3_ and the bottom FASn_0.1_Pb_0.9_I_3_ perovskite (Fig. S17), the spatial evolution of the CBM, VBM, and E_F_ across the depth profile of the films was determined. The corresponding energy-level alignment plots and electronic parameters are presented in Fig. S18 and summarized in Tables S6-S8. Accordingly, the energy-level mismatches at NiO_x_/perovskite and perovskite/C60 interfaces were constructed and depicted in Fig. 5d, e, respectively. Notably, the control perovskite exhibits significant energy-level mismatch at the NiO_x_/perovskite (0.31 eV) interface (Fig. 5d). In contrast, the incorporation of the Sn(OTF)_2_ interlayer simultaneously lowers the VBM of NiO_x_ from − 5.33 to − 5.47 eV and elevates the VBM of the bottom perovskite from − 5.84 to − 5.54 eV for Sn(OTF)_2_ and − 5.53 eV for Sn(OTF)_2_ + PEA. The underlying mechanisms were previously discussed in Figs. 2l and 3g. This modification reduces the energy-level difference at the NiO_x_/perovskite interface to < 0.10 eV, significantly smaller than the 0.31 eV observed in the control device. Furthermore, the F-PEA post-treatment induces formation of 2D perovskite capping layer, enabling the Sn(OTF)_2_ + PEA film to exhibit a substantially reduced energy-level difference of 0.48 eV at the perovskite/C60 interface (Fig. 5e). This value is markedly lower than those of the control (0.70 eV) and Sn(OTF)_2_ (0.67 eV) films. Therefore, with the synergistic contributions of the Sn(OTF)_2_ interlayer and F-PEA post-treatment, the TSP p-i-n PSCs based on the Sn(OTF)_2_ + PEA film achieve optimized energy-level alignment at both NiO_x_/perovskite and perovskite/C60 interfaces. This dual-interface optimization establishes a favorable charge extraction environment while suppressing non-radiative recombination, ultimately yielding the highest device efficiency of 25.6%.

To further understand the notable enhancement in device efficiency, the charge dynamics of the fabricated devices were systematically analyzed. The dependence of V_oc_ and J_sc_ on light intensity (*I*) was investigated to evaluate the charge carrier recombination and extraction efficiency. As presented in Fig. 5f, the Sn(OTF)_2_ + PEA device demonstrates the smallest V_oc_ versus* I* slope (1.31 kT q^−1^) compared to the control (2.05 kT q^−1^) and Sn(OTF)_2_ (1.53 kT q^−1^) devices, suggesting effective suppression of trap-assisted recombination within the Sn(OTF)_2_ + PEA device, which typically results in an enhancement of V_oc_ [47]. The analytical results of J_sc_ dependence on *I* (Fig. S19) indicate that the Sn(OTF)_2_ + PEA device delivers the most ideal α value (0.957), demonstrating improved charge carrier extraction efficiency [48]. In addition, the defect density of the perovskite films was quantitatively estimated using space-charge-limited current (SCLC) technique [49]. Figure 5g displays the dark J-V curves of typical electron-only devices (ITO/SnO_2_/perovskite/[6, 6]-phenyl-C61-butyric acid methyl ester (PCBM)/Ag) fabricated with different films. Notably, the Sn(OTF)_2_ + PEA device yields a trap-filled limit voltage (V_TFL_) of 0.39 V, lower than those of the control (0.64 V) and Sn(OTF)_2_ (0.47 V) devices. As detailedly described and summarized in Table S9, the defect density was determined. The Sn(OTF)_2_ + PEA device exhibits the lowest trap state density (4.21 × 10^15^ cm^−3^), representing a 39.1% reduction compared to the control (6.91 × 10^15^ cm^−3^). While Sn(OTF)_2_ alone passivates buried interface defects, achieving a reduced defect density of 5.07 × 10^15^ cm^−3^ (corresponding to a 26.6% reduction in N_t_), the additional 12.5% reduction from F-PEA treatment confirms effect surface defect passivation. This surface defect passivation originates from fluorine’s high electronegativity, which withdraws electron density from the phenyl ring and enhances the positive charge on the ammonium group (–NH_3_^+^) of the F-PEA^+^ cation. The enhanced charge density strengthens the ionic bonding with negatively charged undercoordinated iodide ions (I^−^) on the perovskite surface. Concurrently, the lone electron pairs on the fluorine atom form stronger coordinate bonds with undercoordinated Pb^2+^ defects [50]. This dual interaction enables more effective defect passivation. Besides, Mott–Schottky analysis of capacitance–voltage (C-V) characteristics was utilized to investigate the influence of the Sn(OTF)_2_ interlayer and F-PEA post-treatment on the built-in potential (V_bi_) of the devices. According to the following equation [51]:

1 $$\frac{1}{{C}^{2}}=\frac{2\left({V}_{bi}-V\right)}{{A}^{2}q\varepsilon {\varepsilon }_{0}N}$$

where C is the depletion-layer capacitance, ɛ is the relative permittivity, ɛ_0_ is the vacuum permittivity, A is the active area of the device, N denotes the density of excited states, and V is the applied voltage, the V_bi_ of the devices increases sequentially from 0.91 V (control) to 1.01 V (Sn(OTF)_2_) and further to 1.04 V (Sn(OTF)_2_ + PEA), as shown in Fig. 5h. This enhancement in V_bi_ is primarily attributed to the optimized energy-level alignment, which promotes efficient charge carrier separation and extraction, thereby contributing to improved V_oc_ in PSCs. Overall, the notable improvement in photovoltaic performance observed for the Sn(OTF)_2_ + PEA devices stem from several key factors, including effective suppression of charge recombination, improved charge extraction efficiency, reduced defect density, and optimized interfacial energy-level alignment.

### Effects of Dual-Interface Modification on Device Stability

**Fig. 6:**
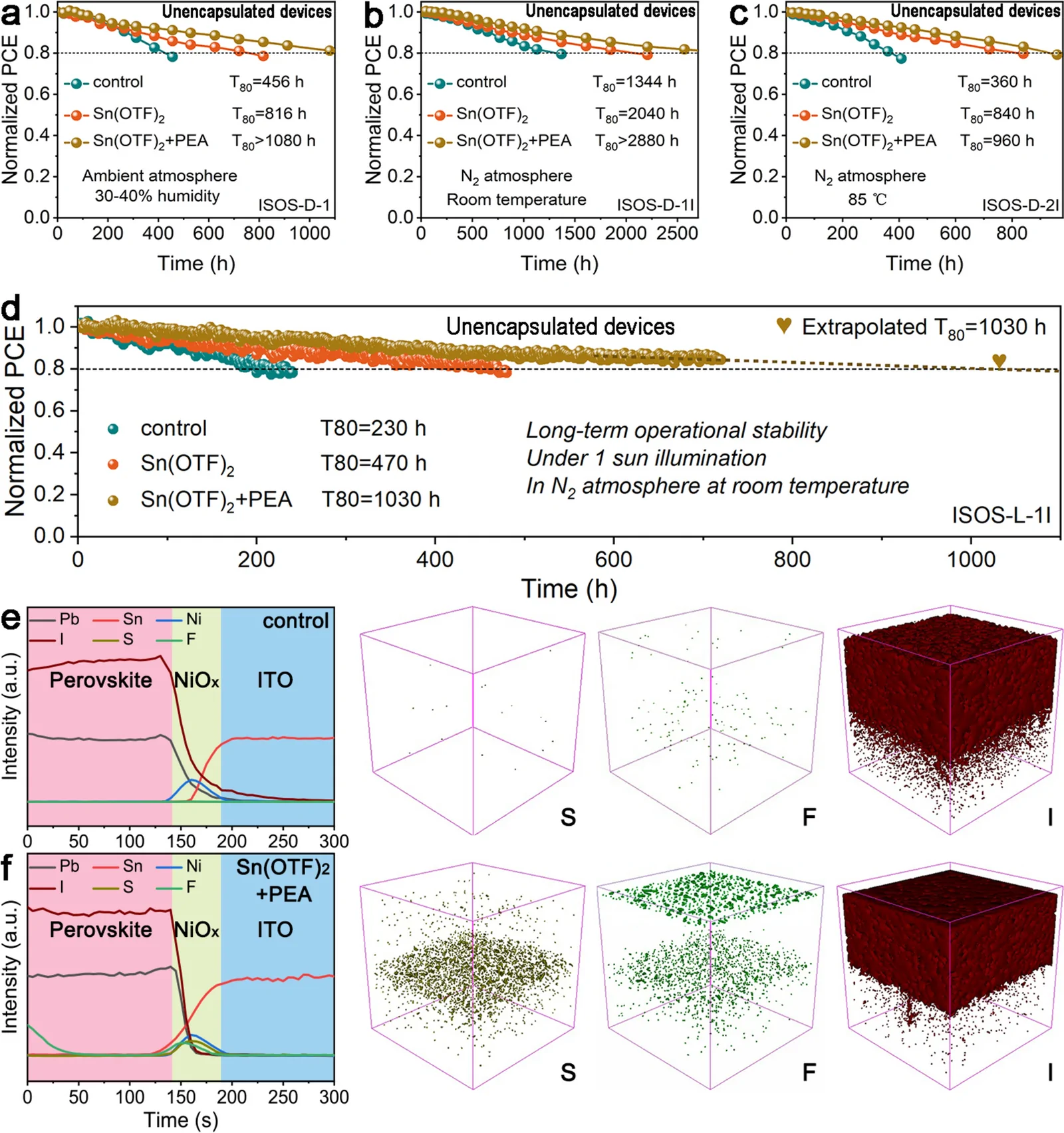
**a** ISOS-D-1 device stability at RH = 30–40%. **b** ISOS-D-1I device stability in N_2_ atmosphere. **c** ISOS-D-2I device stability during storage at 85 °C. **d** ISOS-L-1I device stability under 1 sun illumination. Time-of-flight secondary ion mass spectrometry (TOF–SIMs) depth profiles and corresponding ions distribution of I^⁻^, S^⁻^, and F^⁻^ within the **e** control and **f** Sn(OTF)_2_ + PEA devices

Furthermore, comprehensive stability assessments of unencapsulated devices under various environmental conditions were performed to evaluate the effects of the Sn(OTF)_2_ interlayer and F-PEA post-treatment on device stability. As shown in Fig. 6a, the control device rapidly degraded to 80% of its initial PCE (T_80_ = 456 h) under ambient conditions (30%-40% RH). In contrast, the Sn(OTF)_2_ device exhibited significantly improved stability (T_80_ = 816 h), attributable to its superior perovskite film morphology featuring enlarged crystalline grains and reduced grain boundaries (Fig. 3a), which effectively limit moisture penetration. Notably, after F-PEA post-treatment, the resulting Sn(OTF)_2_ + PEA device achieves an extended T_80_ lifetime exceeding 1080 h under the same conditions. This significant improvement in ambient stability stems primarily from increased hydrophobicity, as evidenced by contact angle measurements (Fig. S20). It should be noted that Sn^2+^ in tin-based perovskites is highly susceptible to oxidation to Sn^4+^, as reported, which induces severe non-radiative recombination and structural degradation, ultimately compromising device efficiency and stability [52]. However, in this work, the oxidation of Sn^2+^ in the Pb–Sn mixed perovskite interlayer was effectively suppressed via three strategies: First, Sn(OTF)_2_ was deposited in an N_2_-filled glovebox to prevent oxidation induced by ambient oxygen. Second, undercoordinated Ni^≥3+^ species on the NiO_x_ surface were effectively passivated by –OTF groups, as discussed earlier. Third, the highly crystalline perovskite film reduces defect-induced reactive oxygen species (ROS) generation, which can oxidize Sn^2+^. To validate the suppressed Sn^2+^ oxidation, unencapsulated perovskite films were exposed to ambient air (35% RH), with the Sn^4+^/Sn^2+^ ratio at the buried interface monitored by XPS. As shown in Fig. S21, the ratio increased marginally from 14.1% (0 h) to 21.0% (168 h). This slow oxidation kinetics indicates the Pb–Sn interlayer possesses enhanced ambient stability. The mild increase aligns with gradual degradation of unencapsulated films, which is unavoidable under ambient conditions but well-controlled here. Besides, the efficiency of unencapsulated devices was recorded under identical ambient conditions (Fig. S22). The PCE decreased slightly from 25.21% (0 h) to 24.7% (168 h). This minor efficiency reduction was primarily attributed to a slight FF decrease (81.9% to 80.8%), whereas the J_sc_ and V_oc_ remained stable. This confirms that the Pb–Sn mixed perovskite interlayer maintains intact energy-level alignment function (critical for J_sc_ and V_oc_), and the degradation primarily originates from minor bulk perovskite deterioration rather than interlayer failure. Additionally, similar improvements in long-term and thermal stability under N_2_ atmosphere were observed (Fig. 6b, c), which are mainly attributed to improved crystallinity and reduced structural defects that effectively inhibit perovskite crystal degradation. Operational stability testing performed under the ISOS-L-1I protocol (under 1 sun illumination in N_2_ atmosphere at room temperature) revealed that the Sn(OTF)_2_ + PEA device maintained exceptional stability, retaining > 84% of initial performance after 720 h. To estimate the T_80_ lifetime, an established lifetime extrapolation method was employed based on the measured data [53]. As shown in Fig. 6d, the extrapolated T_80_ for the Sn(OTF)_2_ + PEA device was projected to reach 1030 h, indicating a twofold improvement relative to the Sn(OTF)_2_ device (T_80_ = 470 h) and a fourfold enhancement compared with the control device (T_80_ = 230 h). This significantly enhanced operational stability arises from two key factors: (1) the F-PEA-induced formation of a protective 2D perovskite capping layer, which effectively reduces residual PbI_2_ clusters (known as degradation centers) [23, 54], and (2) strong chemical interactions between –OTF groups and Pb^2+^ (confirmed by DFT calculations, Fig. 2a) that inhibit ion migration toward NiO_x_.

To elucidate the suppressed ion migration, time-of-flight secondary ion mass spectrometry (TOF–SIMs) analyses were performed. As shown in Fig. 6e, f, the presence and spatial distribution of S elemental signals in the Sn(OTF)_2_ + PEA device confirm the successful introduction of the Sn(OTF)_2_ interlayer at the NiO_x_/perovskite interface. Depth-profiling analysis shows that the control device exhibits an increase in I^−^ intensity and decrease in Pb^2+^ intensity with increasing etching time, further suggesting that the residual PbI_2_ is mainly located at the top surface. Furthermore, the Sn(OTF)_2_ + PEA device reveals a substantial concentration of Sn^2+^ ions within the bottom subsurface region of the perovskite, demonstrating the incorporation of Sn^2+^ ions into the perovskite lattice. Besides, the dual-interface modification in the Sn(OTF)_2_ + PEA device results in F signals being detected at both top and bottom surfaces of the perovskite. Moreover, the control device shows significantly higher concentrations of Pb^2+^ and I^−^ ions at the NiO_x_/perovskite interface compared to the Sn(OTF)_2_ + PEA device, indicating that ion migration is effectively suppressed by the Sn(OTF)_2_ modification. The corresponding three-dimensional I^−^ ion distribution profiles offer more comprehensive visualization of the suppressed ionic migration within the Sn(OTF)_2_ + PEA device.

## Conclusions

In summary, we have demonstrated that TSP perovskite films exhibit vertically gradient distributed residual PbI_2_ clusters forming Schottky heterojunctions with perovskite, leading to interfacial energy-level mismatches within the NiO_x_-based TSP p-i-n PSCs. To address this issue, a vertical interfacial engineering that relies on the synergistic effect between Sn(OTF)_2_ and F-PEA has been developed for precise regulation of residual PbI_2_ clusters. Specifically, the functional –OTF groups in Sn(OTF)_2_ exhibit robust interactions with both NiO_x_ and PbI_2_, which demonstrate dual functionality in enhancing the conductivity of NiO_x_ films while suppressing adverse redox reaction and ion migration from perovskite to the underlying NiO_x_ layer. Besides, the divalent Sn^2+^ ions spontaneously incorporate into the perovskite lattice, forming a Pb–Sn mixed perovskite interlayer that critically optimizes the energy-level alignment at the NiO_x_/perovskite interface. Complementally, F-PEA post-treatment demonstrated efficacy in eliminating residual PbI_2_ clusters through the formation of a 2D perovskite capping layer, which significantly improves perovskite/C60 interfacial energy-level alignment while simultaneously passivating surface defects. With this synergistic collaboration between Sn(OTF)_2_ and F-PEA, the resulting devices deliver a champion PCE of 25.6%, significantly higher than that of Sn(OTF)_2_-only (23.6%) and control (20.5%) devices.

## Supplementary Information

Below is the link to the electronic supplementary material.

Supplementary file1 (DOCX 4945 KB)
