# Supplementary material for: Vertical Interfacial Engineering in Two-Step-Processed Perovskite Films Enabled by Dual-Interface Modification for High-Efficiency p-i-n Solar Cells
Source: Nanomicro Lett. 2026 Jan 5;18:157. doi: 10.1007/s40820-025-02010-w (PMC12765795; doi:10.1007/s40820-025-02010-w)
Supplement: Supplementary file 1 — Supplementary file1 (DOCX 4945 KB) [file 40820_2025_2010_MOESM1_ESM.docx]

Supporting Information for

**Vertical Interfacial Engineering in Two-Step-Processed Perovskite Films Enabled by Dual Interface Modification for High-Efficiency p-i-n Solar Cells**

Wenhao Zhou^1#^, Heng Liu^2,3#^, Haiyan Li^4#^, Weihai Zhang^1,2^,* Hui Li^1^, Xia Zhou^1^, Rouxi Chen^5^, Wenjun Zhang^1^, Tingting Shi^4,^*, Antonio Abate^1^*, and Hsing-Lin Wang^2^*

^1^ School of New Energy, Ningbo University of Technology, Ningbo, 315211, P. R. China

^2^ Department of Materials Science and Engineering, Southern University of Science and Technology, Shenzhen, 518055, P. R. China

^3^ Institute of Materials, Henan Academy of Sciences, Zhengzhou, 450046, P. R. China

^4^ Siyuan Laboratory, Guangzhou Key Laboratory of Vacuum Coating Technologies and New Energy Materials, Department of Physics, Jinan University, Guangzhou, 510632, P. R. China

^5^ School of Innovation and Entrepreneurship, Southern University of Science and Technology, Shenzhen, 518055, P. R. China

#Wenhao Zhou, Heng Liu, and Haiyan Li contributed equally to this work.

*Corresponding authors. E-mail: [zhangwh@nbut.edu.cn](mailto:zhangwh@nbut.edu.cn) (Weihai Zhang); [ttshi@jnu.edu.cn](mailto:ttshi@jnu.edu.cn) (Tingting Shi); [antonio.abate@unina.it](mailto:antonio.abate@unina.it) (Antonio Abate); [wangxl3@sustech.edu.cn](mailto:wangxl3@sustech.edu.cn) (Hsing-Lin Wang)

**Supplementary Figures and Tables**


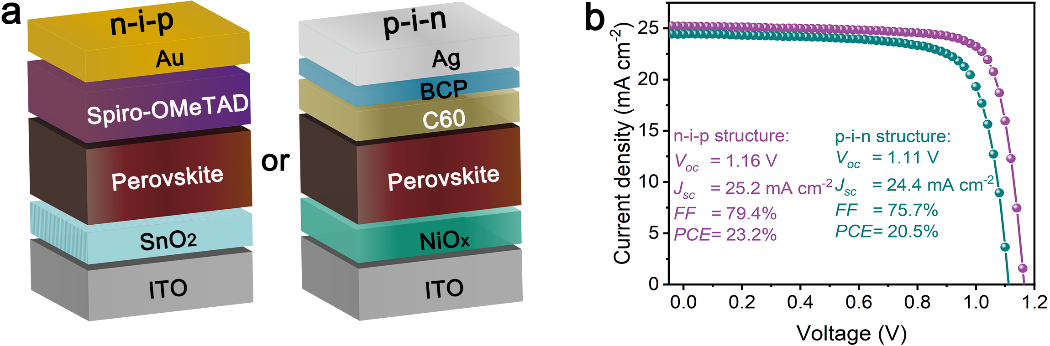


**Fig. S1** **a** Schematic structure of the regular n-i-p and inverted p-i-n devices. **b** J-V curves of the TSP n-i-p and p-i-n devices


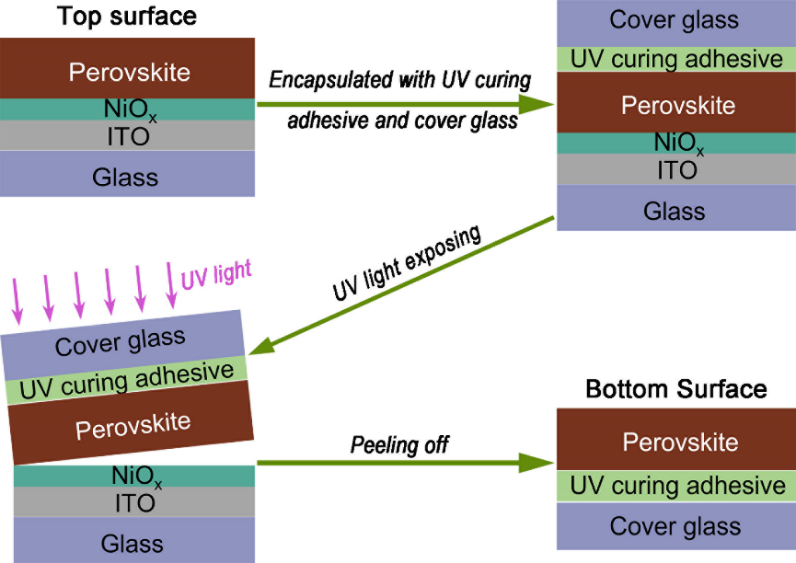


**Fig. S2** Exfoliation process of the perovskite film

Note: The detailed exfoliation process as follows: 1) The as-deposited perovskite films were encapsulated by dispensing a drop of UV-curable adhesive and then covered with a clean glass. 2) After encapsulating the sample, it was exposed to 365 nm UV light for 10 minutes to cure the UV-curable adhesive. 3) The ITO substrate was then gently peeled off to expose the bottom surface of the perovskite film for investigation.


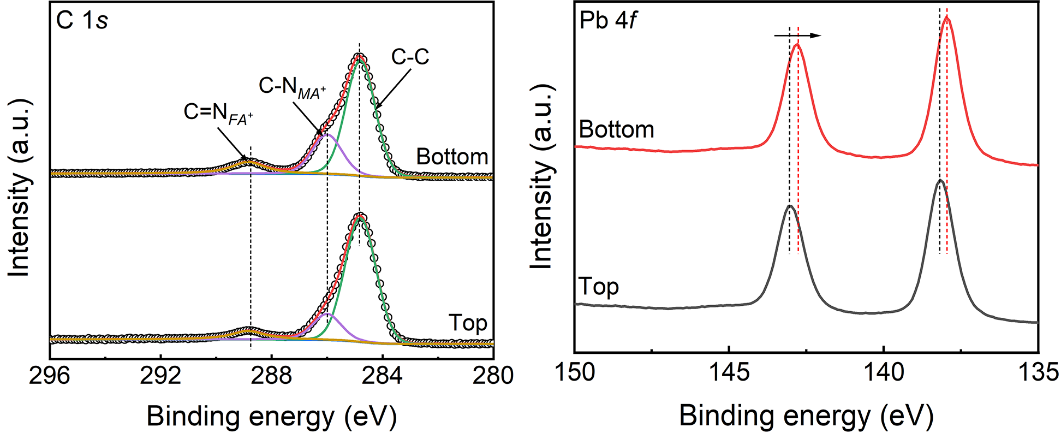


**Fig. S3** XPS C 1s and Pb 4f core spectra of the top and bottom sides of the TSP film


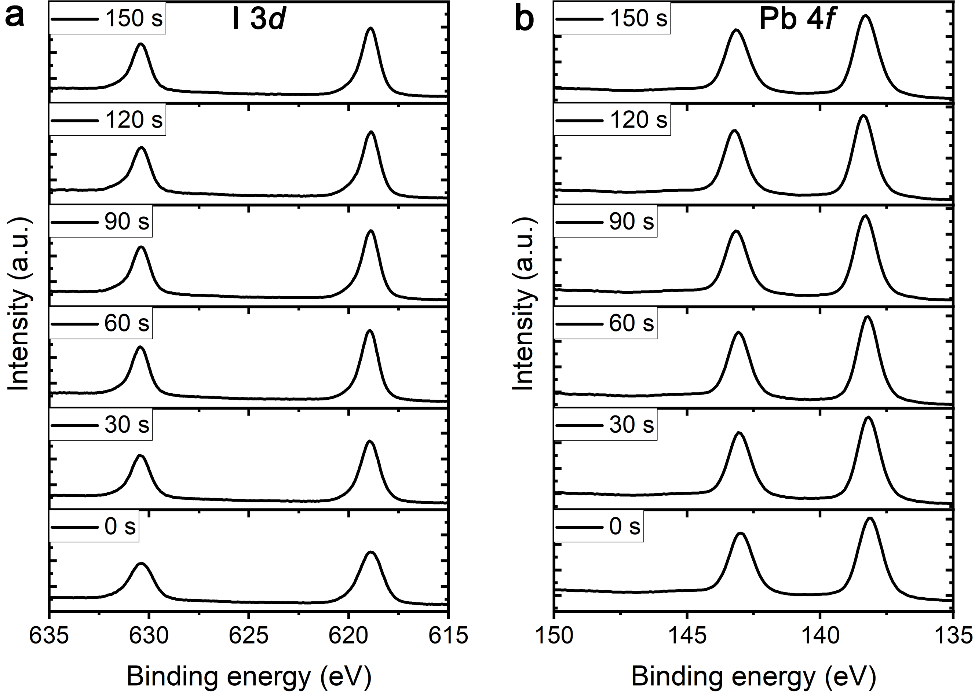


**Fig. S4** Depth-profiled **a** I 3d and **b** Pb 4f core spectra of the TSP film with varying etching time

Note: The I/Pb ratio of the TSP perovskite film with different etching time can be estimated according to the equation: $N_{I}/N_{Pb}=\left( A_{I}/S_{I} \right)/\left( A_{Pb}/S_{Pb} \right)$, where $A_{I}$ and $A_{Pb}$ represent the I 3d and Pb 4f peak areas, respectively, and $S_{I}$ (5.337) and $S_{Pb}$ (6.968) denote the sensitivity factors for I and Pb. The detailed parameteres are summarized in Table S1.


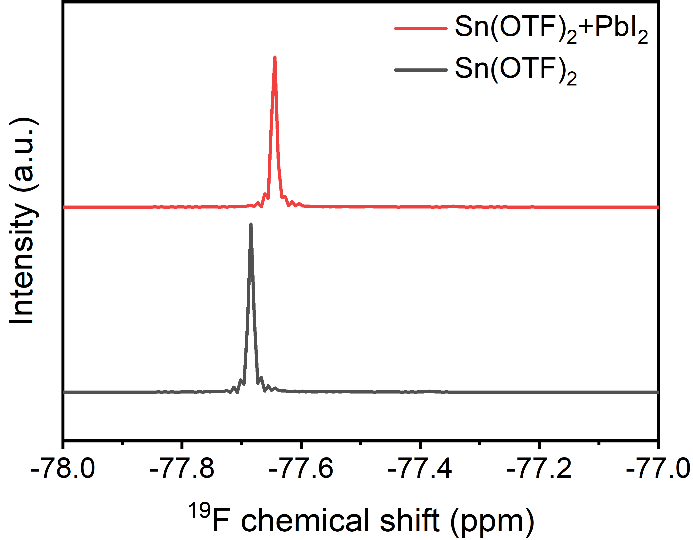


**Fig. S5** ^19^F NMR spectra of pure Sn(OTF)_2_ and Sn(OTF)_2_+PbI_2_ samples in deuterated DMSO-*d*_6_ solution

Note: The characteristic resonance signal of F in pure Sn(OTF)_2_ reveals upshift of resonance peak for the Sn(OTF)_2_+PbI_2_ sample, verifying the interaction between -CF_3_ groups and PbI_2_.


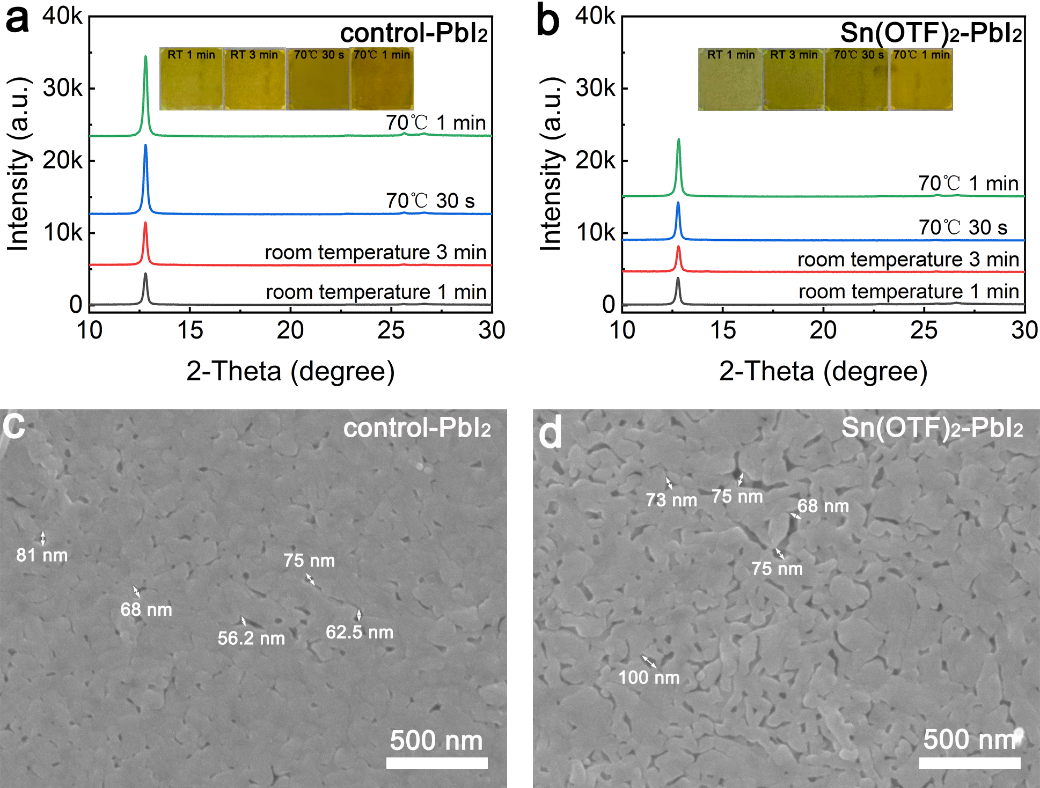


**Fig. S6** **a, b** XRD patterns and **c, d** SEM images of PbI_2_ films deposited on bare NiO_x_ (control) and NiO_x_/Sn(OTF)_2_ (Sn(OTF)_2_) substrates

Note: The inset photos suggest that the control-PbI_2_ films exhibit a significantly faster color transition from semitransparent yellow to deep yellow compared to the Sn(OTF)_2_-PbI_2_ films. This visual observation directly suggests that the control-PbI_2_ films undergo crystallization at a higher rate than the Sn(OTF)_2_-PbI_2_ films. Consistently, the corresponding XRD results reveal that under identical experimental conditions, the control-PbI_2_ films display substantially stronger diffraction intensities relative to the Sn(OTF)_2_-PbI_2_ films, confirming the faster crystallization rate of the control-PbI_2_ films. The SEM results suggest that the Sn(OTF)_2_-PbI_2_ film demonstrates a more mesoporous morphology than that of the control-PbI_2_ film, indicating that Sn(OTF)_2_ interlayer interact with PbI_2_, thus retarding its crystallization. This more mesoporous PbI_2_ film is beneficial for ammonium salt penetration, facilitating perovskite crystallization.


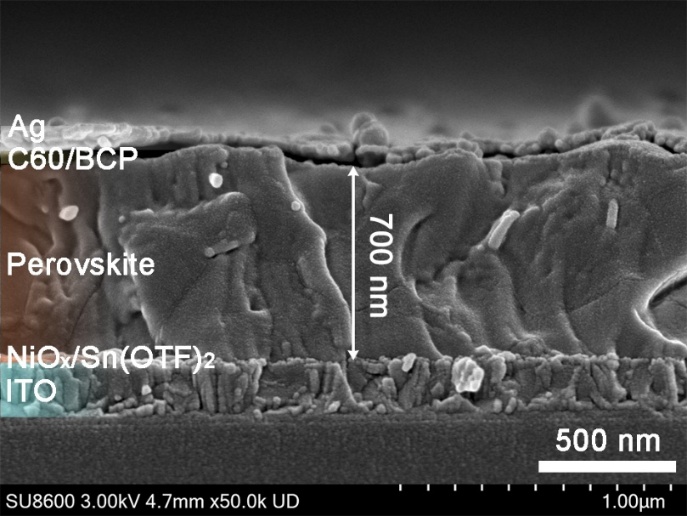


**Fig. S7** Cross-sectional SEM image of the device

Note: According to the scale bar, the thickness of the perovskite film is approximately 700 nm.


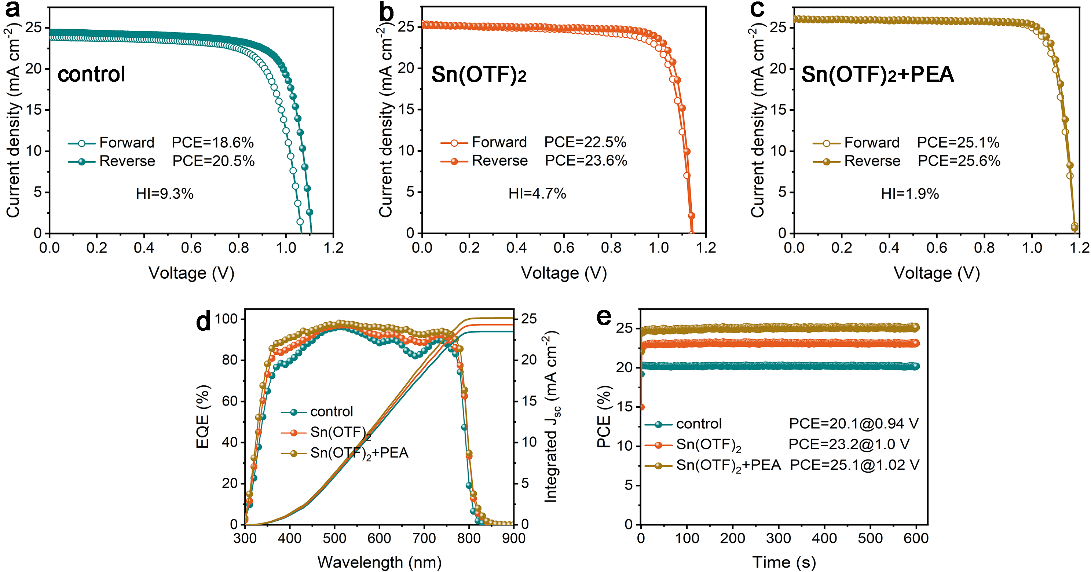


**Fig. S8** J-V curves obtained from reverse (V_oc_ to J_sc_) and forward (J_sc_ to V_oc_) scans for the **a** control, **b** Sn(OTF)_2_, and **c** Sn(OTF)_2_+PEA devices. **d** EQE spectra and **e** stabilized power outputs of the champion devices based on the control, Sn(OTF)_2_, and Sn(OTF)_2_+PEA films

Note: Hysteresis studies based on H-index: $HI=\left( {PCE}_{reverse}-{PCE}_{forward} \right)/{{PCE}_{reverse}}$, where PCE_reverse_ and PCE_forward_ are power conversion efficiency of devices for reverse and forward scan, respectively, reveal that Sn(OTF)_2_+PEA device delivers the smallest HI value (1.9%) compared to the control (9.3%) and Sn(OTF)_2_ (4.7%) devices, which is mainly attributed to a more favorable charge extraction environment within the Sn(OTF)_2_+PEA device.


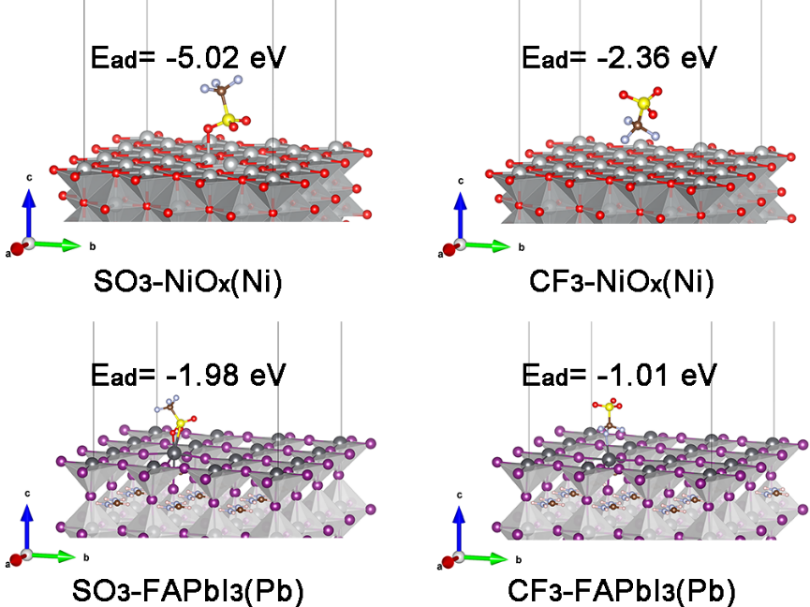


**Fig. S9** Models and adsorption energies of SO_3_-NiO_x_(Ni), CF_3_-NiO_x_(Ni), SO_3_-FAPbI_3_(Pb), and CF_3_-FAPbI_3_(Pb), respectively


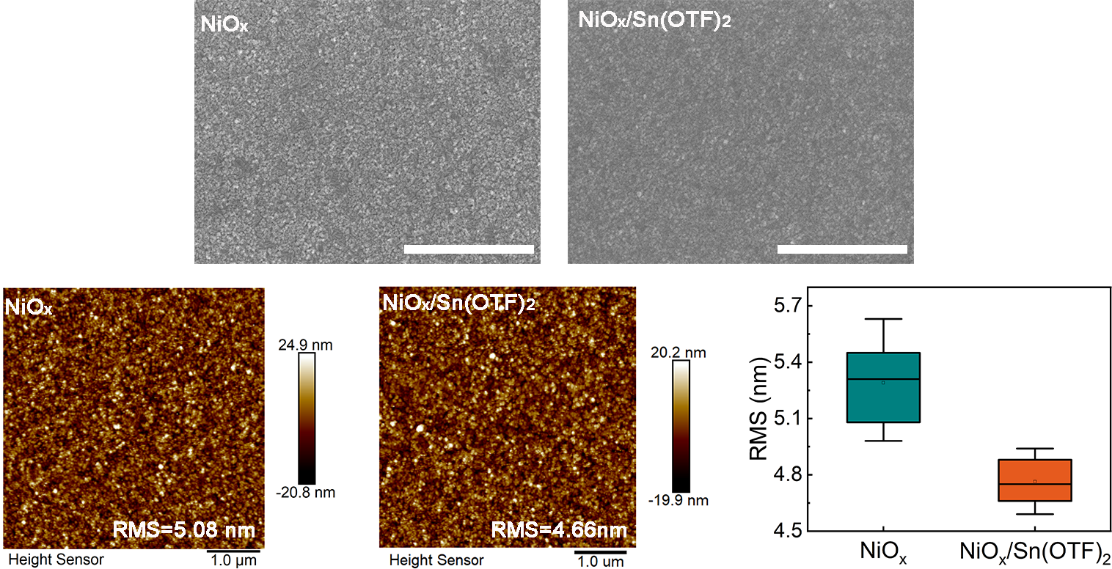


**Fig. S10** SEM and AFM images of the bare NiO_x_ and NiO_x_/Sn(OTF)_2_ films. The scale bar is 1 μm. Statistical RMS values for different films. A total of 5 different random positions (5 × 5 μm^2^ per position) for each sample were counted

Note: The Sn(OTF)_2_ modification does not alter the morphology of NiO_x_ films. While, a reduction in RMS roughness from 5.29 ± 0.34 nm (bare NiO_x_) to 4.76 ± 0.18 nm (NiO_x_/Sn(OTF)_2_) can be observed.


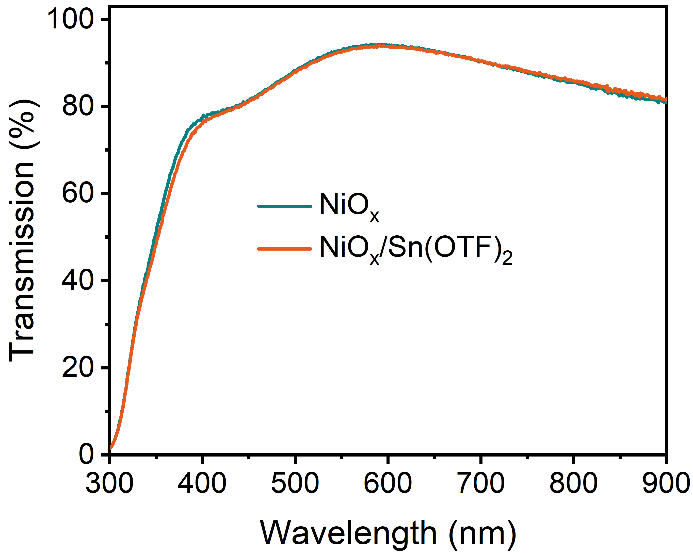


**Fig. S11** Optical transmission spectra of the bare NiO_x_ and NiO_x_/Sn(OTF)_2_ films


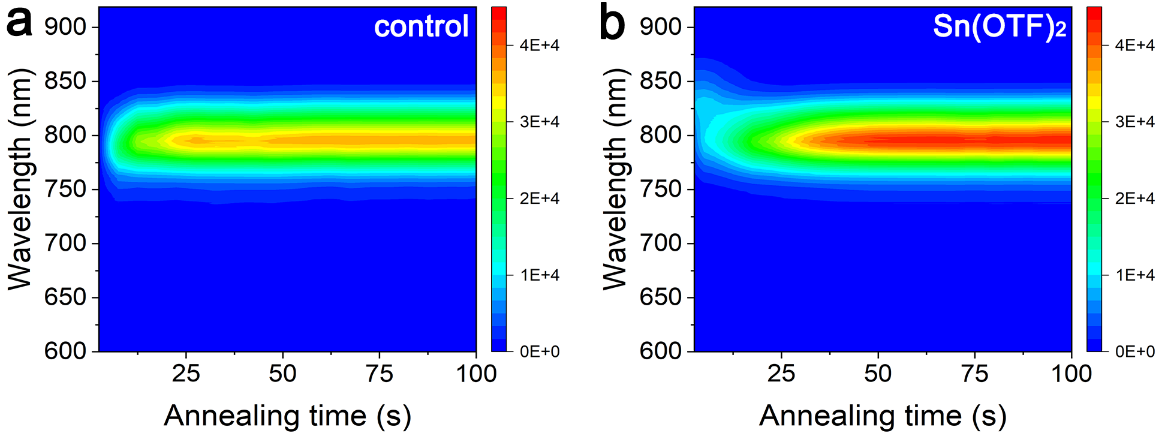


**Fig. S12** In-situ PL spectra of control and Sn(OTF)_2_ perovskite films as a function of different annealing time


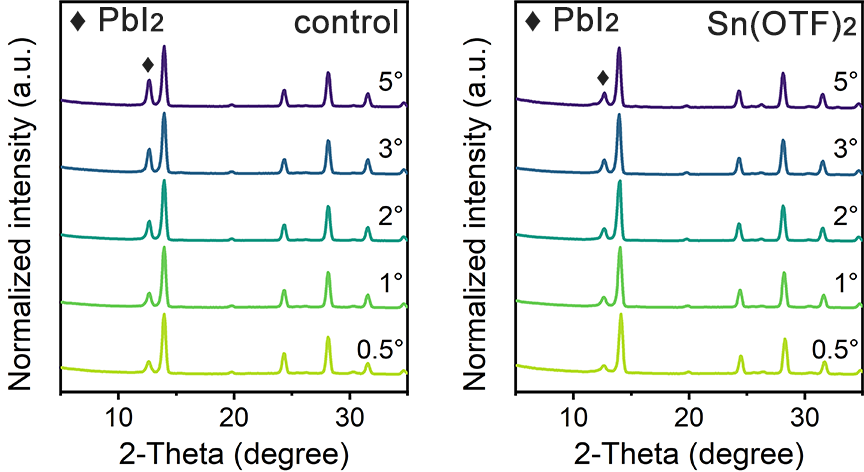


**Fig. S13** GIXRD spectra of the control and Sn(OTF)_2_ films acquired from the bottom surface


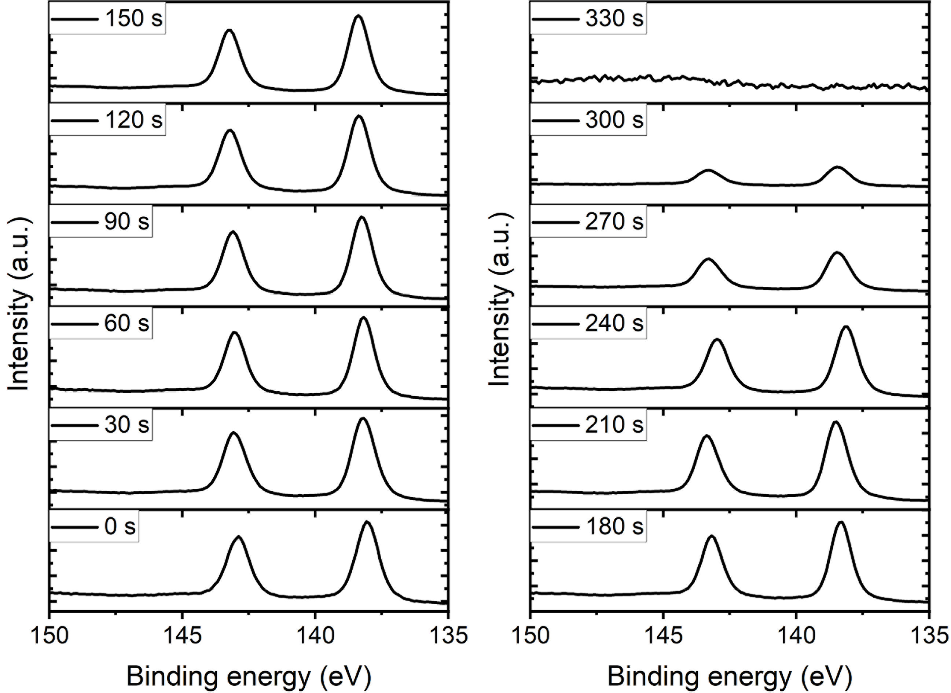


**Fig. S14** Depth-profiled Pb 4f core spectra of the Sn(OTF)_2_ film with varying etching time

Note: It can be seen that after 330 seconds of etching, the Pb 4f peak disappeared, indicating complete etching through the perovskite film. By combining the thickness (700 nm) measured via cross-sectional SEM with the etching duration, the etching rate was calculated to be 2.1 nm/s.


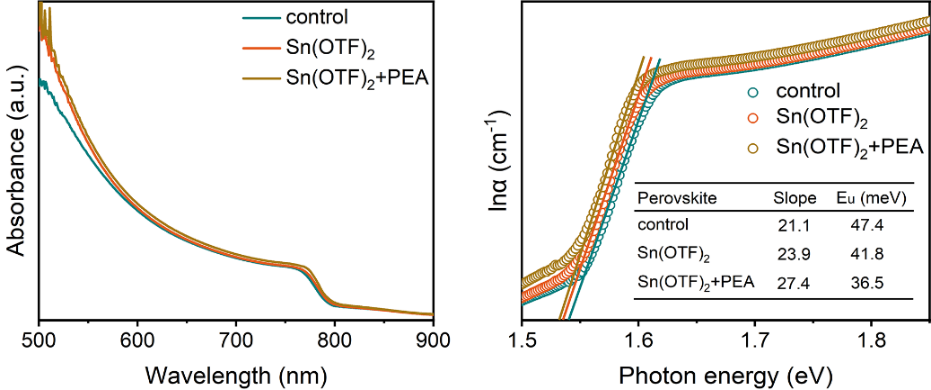


**Fig. S15** UV-vis absorption spectra and Urbach energy of different perovskite film

Note: The Urbach energy was calculated from the UV-Vis absorption spectra with equation $\ln\alpha=\ln\alpha_{0}+\left( {hv}/{E_{u}} \right)$, where α_0_ is a constant, α is the absorption coefficient, hυ is the photon energy, and Eu is the Urbach energy. A smaller E_u_ is highly desirable for semiconductor devices, indicative of highly crystallized film with less impurities.


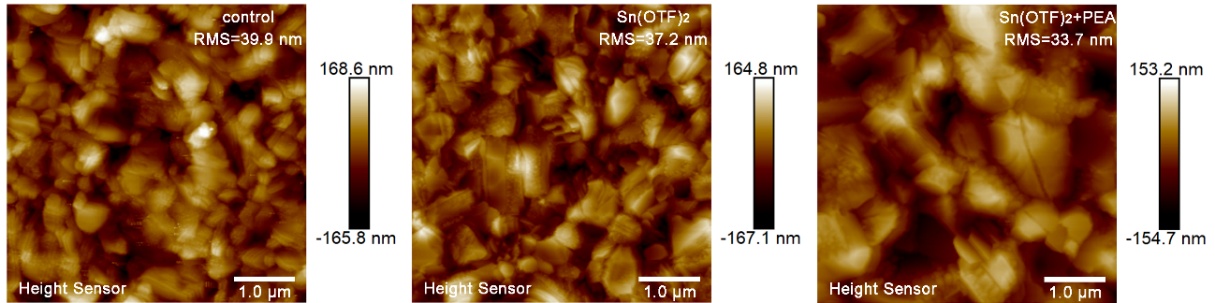


**Fig. S16** AFM images of different perovskite films

Note: The RMS values of the control, Sn(OTF)_2_ and Sn(OTF)_2_+PEA films are 39.9, 37.2, and 33.7 nm, respectively. The Sn(OTF)_2_+PEA film exhibits the smallest surface roughness, which is critical for achieving high device performance.


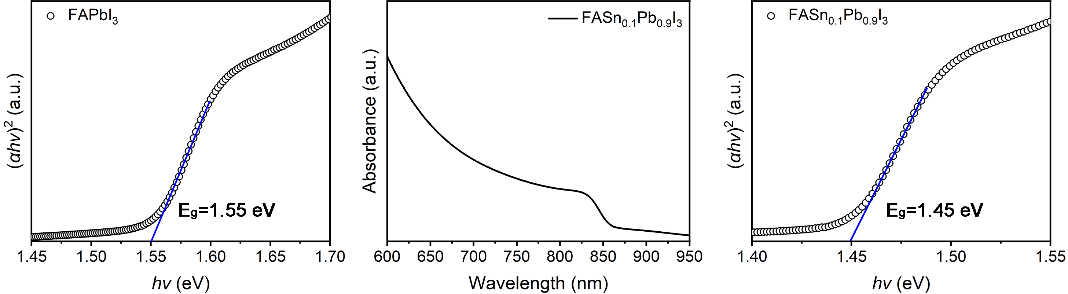


**Fig. S17** Tauc’s plots of the FAPbI_3_ and FASn_0.1_Pb_0.9_I_3_ perovskite films

Note: The Tauc’s plots were derived from the UV-Vis absorption spectra with equation $\left( \alpha h\nu\right)^{2}=A\left( {h\upsilon-E}_{g} \right)$, where α is the absorption coefficient, hυ is the photon energy, and E_g_ is the bandgap of the perovskite film. Accordingly, the bandgaps of the FAPbI_3_ and FASn_0.1_Pb_0.9_I_3_ perovskites were determined to be 1.55 and 1.45 eV, respectively.


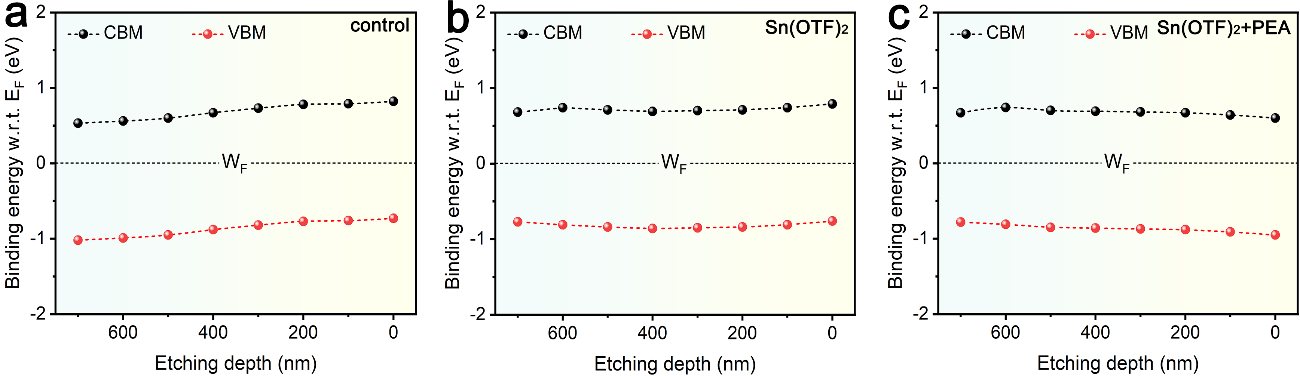


**Fig. S18** Energy level alignment plots of the **a** control, **b** Sn(OTF)_2_, and **c** Sn(OTF)_2_+PEA films


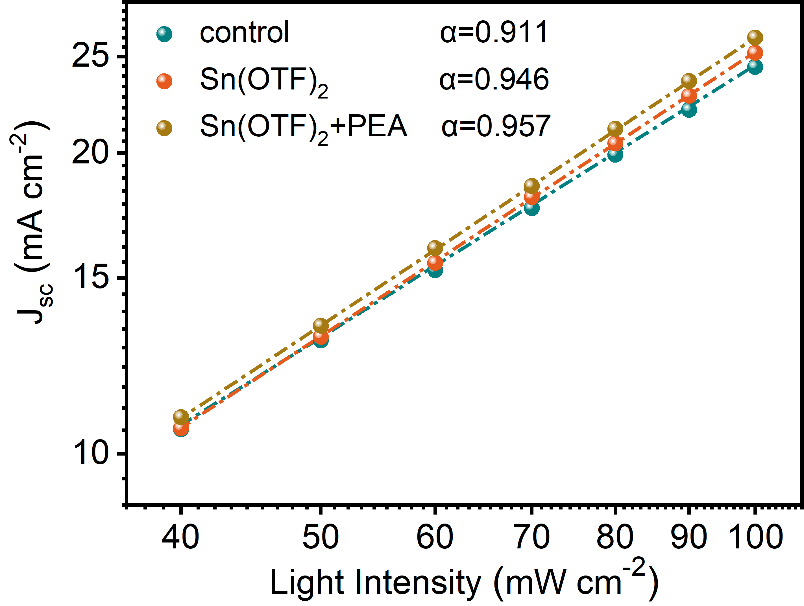


**Fig. S19** J_sc_ response under different light intensities

Note: The Sn(OTF)_2_+PEA device delivers a most ideal α value of 0.957 compared to the control (0.911) and Sn(OTF)_2_ (0.946) devices, demonstrating improved charge carrier extraction efficiency.


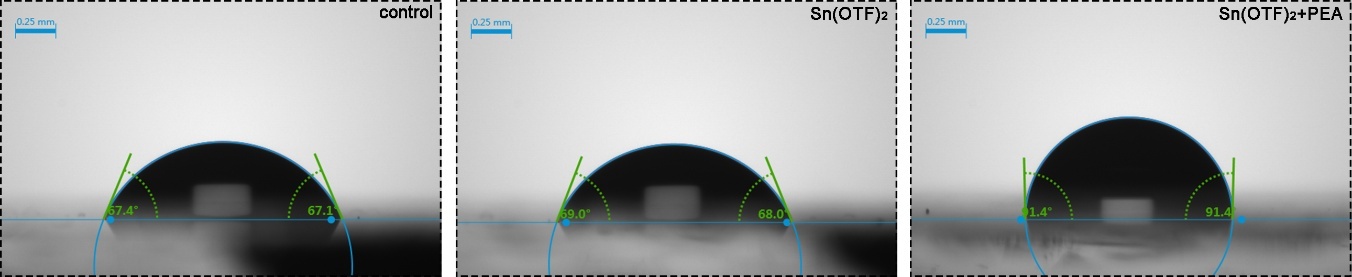


**Fig. S20** The contact angle of control, Sn(OTF)_2_, and Sn(OTF)_2_+PEA films

Note: It can be seen that the Sn(OTF)_2_+PEA film exhibits a contact angle of 91.4°, much larger than that of control (67.4°) and Sn(OTF)_2_ (69°) films. The results suggest that after F-PEA post-treatment, the resulting Sn(OTF)_2_+PEA film demonstrates a more hydrophobic surface, which is essential for the enhancement of long term stability of the resulting device.


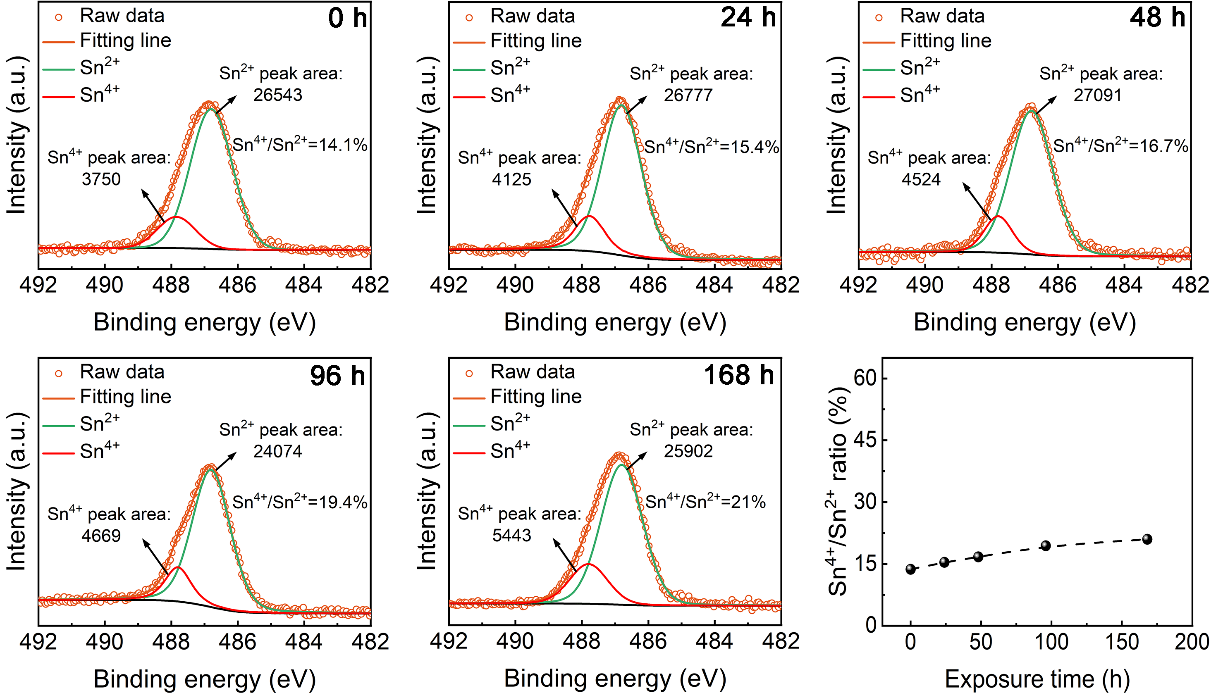


**Fig. S21** Sn 3d_5/2_ XPS core spectra derived from the bottom surface of the perovskite film as a function of ambient exposure time

Note: It can be seen that after 168 h exposure, the Sn^4+^/Sn^2+^ ratio only increased slightly from 14.1 to 21.0%. This slow oxidation rate indicates the Pb-Sn interlayer has strong ambient resilience. The mild increase aligns with the gradual degradation of unencapsulated perovskite films, which is unavoidable in ambient conditions but well-controlled here.


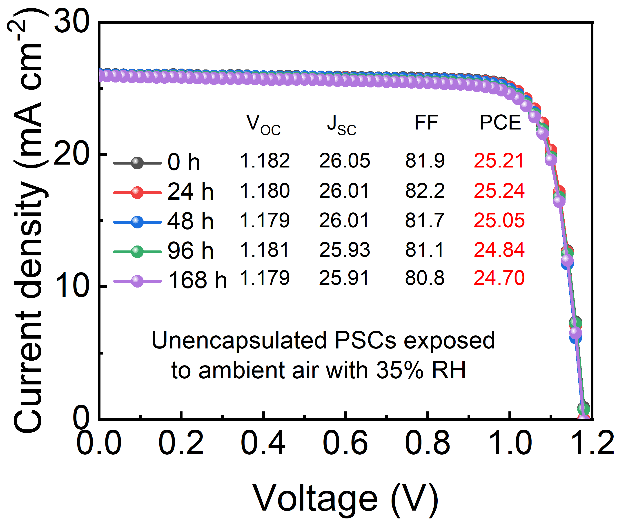


**Fig. S22** J-V curves and corresponding photovoltaic parameters of the unencapsulated PSCs a function of ambient exposure time

Note: It can be seen that after 168 h exposure, the PCE of the device decreased slightly from 25.21%to 24.7%. The minor efficiency loss was primarily attributed to a small drop in FF (from 81.9% to 80.8%), not J_SC_ or V_OC_. This confirms the interlayer’s energy level alignment function (critical for J_SC_ and V_OC_) remains intact, and the decay stems from trivial bulk perovskite degradation, not interlayer failure.

**Table S1** I/Pb ratio of the TSP perovskite film with different etching time

| Etching time (s) | A_I_ | A_Pb_ | S_I_ | S_Pb_ | I/Pb ratio |
| --- | --- | --- | --- | --- | --- |
| 0 | 1616816 | 824578 | 5.337 | 6.968 | 2.56 |
| 30 | 1624230 | 818762 | 5.337 | 6.968 | 2.59 |
| 60 | 1643074 | 812574 | 5.337 | 6.968 | 2.64 |
| 90 | 1678798 | 808763 | 5.337 | 6.968 | 2.71 |
| 120 | 1712927 | 801578 | 5.337 | 6.968 | 2.79 |
| 150 | 1728758 | 797538 | 5.337 | 6.968 | 2.83 |

**Table S2** Photovoltaic parameters of champion PSCs based on different perovskite films

| Device | Scan direction | Voltage (V) | Current density (mA cm-2) | FF (%) | PCE (%) | HI (%) |
| --- | --- | --- | --- | --- | --- | --- |
| control | Forward | 1.06 | 23.9 | 73.1 | 18.6 | 9.3 |
|  | Reverse | 1.11 | 24.4 | 75.7 | 20.5 |  |
| Sn(OTF)_2_ | Forward | 1.14 | 25.4 | 77.5 | 22.5 | 4.7 |
|  | Reverse | 1.14 | 25.2 | 81.8 | 23.6 |  |
| Sn(OTF)_2_+PEA | Forward | 1.18 | 26 | 81.5 | 25.1 | 1.9 |
|  | Reverse | 1.18 | 26.1 | 82.8 | 25.6 |  |

**Table S3** Conductivity of bare NiO_x_ and NiO_x_/Sn(OTF)_2_ films

| Sample | Thickness (nm) | Area (cm^2^) | slope | Conductivity (S·cm^-1^) |
| --- | --- | --- | --- | --- |
| NiO_x_ | 40 | 0.04 | 0.2 | 2🞨10^-5^ |
| NiO_x_/Sn(OTF)_2_ | 40 | 0.04 | 0.26 | 2.6🞨10^-5^ |

Note: The conductivity of the films were calculated according to the equation: σ=D/(SR), where σ is the conductivity, D is the film thickness, S is the effective area, and R is the resistance. Based on the I-V curve, R can be extracted as 1/slope.

**Table S4** UPS parameters of the bare NiO_x_ and NiO_x_/Sn(OTF)_2_ films

| Sample | E_cutoff_ (eV) | E_onset_ (eV) | VBM (eV) | E_F_ (eV) |
| --- | --- | --- | --- | --- |
| NiO_x_ | 16.60 | 0.71 | 5.33 | 4.62 |
| NiO_x_/Sn(OTF)_2_ | 16.43 | 0.68 | 5.47 | 4.79 |

**Table S5** Fitted TRPL parameters of the different perovskite films

| Perovskite | A_1_ | τ_1_ (ns) | A_2_ | τ_2_ (ns) | τ_ave_ (ns) |
| --- | --- | --- | --- | --- | --- |
| control | 0.406 | 181.45 | 0.473 | 386.8 | 327.9 |
| Sn(OTF)_2_ | 0.59 | 240.5 | 0.256 | 792.4 | 565.2 |
| Sn(OTF)_2_+PEA | 0.696 | 337.13 | 0.225 | 1140 | 756.6 |

Note: The fitting biexponential decay function was $y=y_{0}+A_{1}exp\left( -\frac{t}{\tau_{1}} \right)+A_{2}exp\left( -\frac{t}{\tau_{2}} \right)$.

**Table S6** UPS parameters of the control perovskite film with different etching depths

| Depth [nm] | E_cutoff_ (eV) | E_onset_ (eV) | VBM (eV) | E_g_ (eV) | CBM (eV) | E_F_ (eV) |
| --- | --- | --- | --- | --- | --- | --- |
| 700 | 16.4 | 1.02 | -5.84 | 1.55 | -4.29 | 4.82 |
| 600 | 16.43 | 0.99 | -5.78 | 1.55 | -4.23 | 4.79 |
| 500 | 16.46 | 0.95 | -5.71 | 1.55 | -4.16 | 4.76 |
| 400 | 16.46 | 0.88 | -5.64 | 1.55 | -4.09 | 4.76 |
| 300 | 16.45 | 0.82 | -5.59 | 1.55 | -4.04 | 4.77 |
| 200 | 16.47 | 0.77 | -5.52 | 1.55 | -3.97 | 4.75 |
| 100 | 16.5 | 0.76 | -5.48 | 1.55 | -3.93 | 4.72 |
| 0 | 16.53 | 0.73 | -5.42 | 1.55 | -3.87 | 4.69 |

**Table S7** UPS parameters of the Sn(OTF)_2_ perovskite film with different etching depths

| Depth [nm] | E_cutoff_ (eV) | E_onset_ (eV) | VBM (eV) | E_g_ (eV) | CBM (eV) | E_F_ (eV) |
| --- | --- | --- | --- | --- | --- | --- |
| 700 | 16.45 | 0.77 | -5.54 | 1.45 | -4.09 | 4.77 |
| 600 | 16.43 | 0.81 | -5.6 | 1.55 | -4.05 | 4.79 |
| 500 | 16.42 | 0.84 | -5.64 | 1.55 | -4.09 | 4.8 |
| 400 | 16.41 | 0.86 | -5.67 | 1.55 | -4.12 | 4.81 |
| 300 | 16.41 | 0.85 | -5.66 | 1.55 | -4.11 | 4.81 |
| 200 | 16.4 | 0.84 | -5.66 | 1.55 | -4.11 | 4.82 |
| 100 | 16.41 | 0.81 | -5.62 | 1.55 | -4.07 | 4.81 |
| 0 | 16.4 | 0.76 | -5.58 | 1.55 | -4.03 | 4.82 |

**Table S8** UPS parameters of the Sn(OTF)_2_+PEA perovskite film with different etching depths

| Depth [nm] | E_cutoff_ (eV) | E_onset_ (eV) | VBM (eV) | E_g_ (eV) | CBM (eV) | E_F_ (eV) |
| --- | --- | --- | --- | --- | --- | --- |
| 700 | 16.47 | 0.78 | -5.53 | 1.45 | -4.08 | 4.75 |
| 600 | 16.42 | 0.81 | -5.61 | 1.55 | -4.06 | 4.80 |
| 500 | 16.42 | 0.85 | -5.65 | 1.55 | -4.10 | 4.80 |
| 400 | 16.41 | 0.86 | -5.67 | 1.55 | -4.12 | 4.81 |
| 300 | 16.41 | 0.87 | -5.68 | 1.55 | -4.13 | 4.81 |
| 200 | 16.4 | 0.88 | -5.70 | 1.55 | -4.15 | 4.82 |
| 100 | 16.39 | 0.91 | -5.74 | 1.55 | -4.19 | 4.83 |
| 0 | 16.38 | 0.95 | -5.79 | 1.55 | -4.24 | 4.84 |

**Table S9** SCLC parameters for different perovskite films

| Perovskite | Dielectric factor | Vacuum permittivity (F m^-1^) | Thickness (nm) | Elementary charge | V_TFL_ (V) | Trap density (cm^-3^) |
| --- | --- | --- | --- | --- | --- | --- |
| control | 48 | 8.85×10^-12^ | 700 | 1.6×10^-19^ | 0.64 | 6.91×10^15^ |
| Sn(OTF)_2_ | 48 | 8.85×10^-12^ | 700 | 1.6×10^-19^ | 0.47 | 5.07×10^15^ |
| Sn(OTF)_2_+PEA | 48 | 8.85×10^-12^ | 700 | 1.6×10^-19^ | 0.39 | 4.21×10^15^ |

Note: The trap density of the perovskite films can be calculated through the following equation:

$N_{t}=\frac{{2V}_{TFL}\varepsilon_{r}\varepsilon_{0}}{{eL}^{2}}$ (S1)

where ε_r_ is the relative dielectric constant of perovskite, which is approximately 48, ε_0_ is the vacuum permittivity, *e* is the elementary charge, and *L* is the thickness of the perovskite film, which is approximately 700 nm. Accordingly, the defect density was determined to be 6.91×10^15^, 5.07×10^15^, and 4.21×10^15^ cm^-3^ for the control, Sn(OTF)_2_, and Sn(OTF)_2_+PEA films, respectively.
